# Overview of Biologically Active Nucleoside Phosphonates

**DOI:** 10.3389/fchem.2020.616863

**Published:** 2021-01-08

**Authors:** Elisabetta Groaz, Steven De Jonghe

**Affiliations:** ^1^Medicinal Chemistry, Rega Institute for Medical Research, KU Leuven, Leuven, Belgium; ^2^Laboratory of Virology and Chemotherapy, Rega Institute for Medical Research, KU Leuven, Leuven, Belgium

**Keywords:** nucleoside phosphonate, antivirals, anticancer drugs, antibacterials, antiparasitic agents, purinergic signaling

## Abstract

The use of the phosphonate motif featuring a carbon-phosphorous bond as bioisosteric replacement of the labile P–O bond is widely recognized as an attractive structural concept in different areas of medicinal chemistry, since it addresses the very fundamental principles of enzymatic stability and minimized metabolic activation. This review discusses the most influential successes in drug design with special emphasis on nucleoside phosphonates and their prodrugs as antiviral and cancer treatment agents. A description of structurally related analogs able to interfere with the transmission of other infectious diseases caused by pathogens like bacteria and parasites will then follow. Finally, molecules acting as agonists/antagonists of P2X and P2Y receptors along with nucleotidase inhibitors will also be covered. This review aims to guide readers through the fundamentals of nucleoside phosphonate therapeutics in order to inspire the future design of molecules to target infections that are refractory to currently available therapeutic options.

## General Introduction

Natural nucleosides are the building blocks of RNA and DNA and are composed of a nucleobase and a sugar moiety. Nucleoside analogs are structurally modified in either the sugar and/or base moiety. In order to become biologically active, nucleoside analogs need, very often, to be converted to their phosphorylated counterparts furnishing nucleotide analogs. Many nucleoside analogs are not phosphorylated effectively in cells, with the first phosphorylation often being the rate-limiting step. In principle, the administration of nucleoside monophosphate analog allows the first phosphorylation step in the nucleoside activation process to be skipped, therefore bypassing the rate-limiting step in the conversion. However, the phosphorus-oxygen bond of a nucleoside monophosphate is susceptible to enzymatic hydrolysis by phosphatases and phosphodiesterases and hence nucleoside-phosphate analogs cannot be pursued as drug candidates. It has encouraged medicinal chemists to synthesize nucleoside phosphonates as mimics of nucleoside phosphates. A major advantage of nucleoside phosphonates is their chemical and metabolic stability, as the phosphorus-carbon bond is not susceptible to enzymatic hydrolysis.

Various nucleoside phosphonates have been prepared as phosphate isosteres. Examples include deoxy nucleoside phosphonates in which the oxygen atom in α-position of the phosphorus is either removed or replaced by a methylene moiety. The phosphonomethoxy (P–C–O) functionality, in which the 5′-oxygen and 5′-carbon are swapped from place, emerged as the most promising isoster of the phosphonooxymethyl group (P–O–C) of the naturally occurring nucleoside-monophosphate. Its success is due to the fact that it is isopolar and isosteric with the phosphate group. Another important characteristic of nucleoside phosphonates is their ability to function as substrates for various kinases and hence to undergo enzymatic phosphorylation that converts them into diphospho phosphonate analogs. This is especially important for antiviral and antitumoral drugs, as in these cases, the pharmacologically active species is a nucleoside triphosphate analog.

Although metabolically stable, phosphonates are negatively charged at physiological pH, and hence, are not able to penetrate the lipid-rich cell membrane, which hampers their cellular activity (either antiviral, antitumoral, antibacterial, or antiparasitic). A variety of phosphonate prodrug strategies have been developed, which are mainly pursued in the antiviral area, but recently attracted attention in other therapeutic fields as the antibacterials and antiparasitics.

Previous reviews dealing with nucleoside phosphonates are mainly focused on their antiviral properties (Mackman and Cihlar, 2011; Pertusati et al., 2012; Baszczynski and Janeba, 2013; Macchi et al., 2015) or synthetic procedures (Pradere et al., 2014; Shen and Hong, 2019). Although nucleoside phosphonates have been historically mainly pursued because of their antiviral properties, they exhibit a wide range of other biological activities, such as antitumoral, antibacterial, and antiparasitic. In addition, nucleoside phosphonates are known to interfere in the purinergic signaling process. This review focuses on different biological properties associated with nucleoside phosphonates and is organized according to the therapeutic area or biological target. Only nucleoside phosphonates with demonstrated biological activity, either *in vitro* or *in vivo*, are highlighted in this review. For a full discussion of synthesis and structure-activity relationships (SAR), the reader is referred to the original research papers.

## Anti-Herpesviral Nucleoside Phosphonates

**Figure 1:**
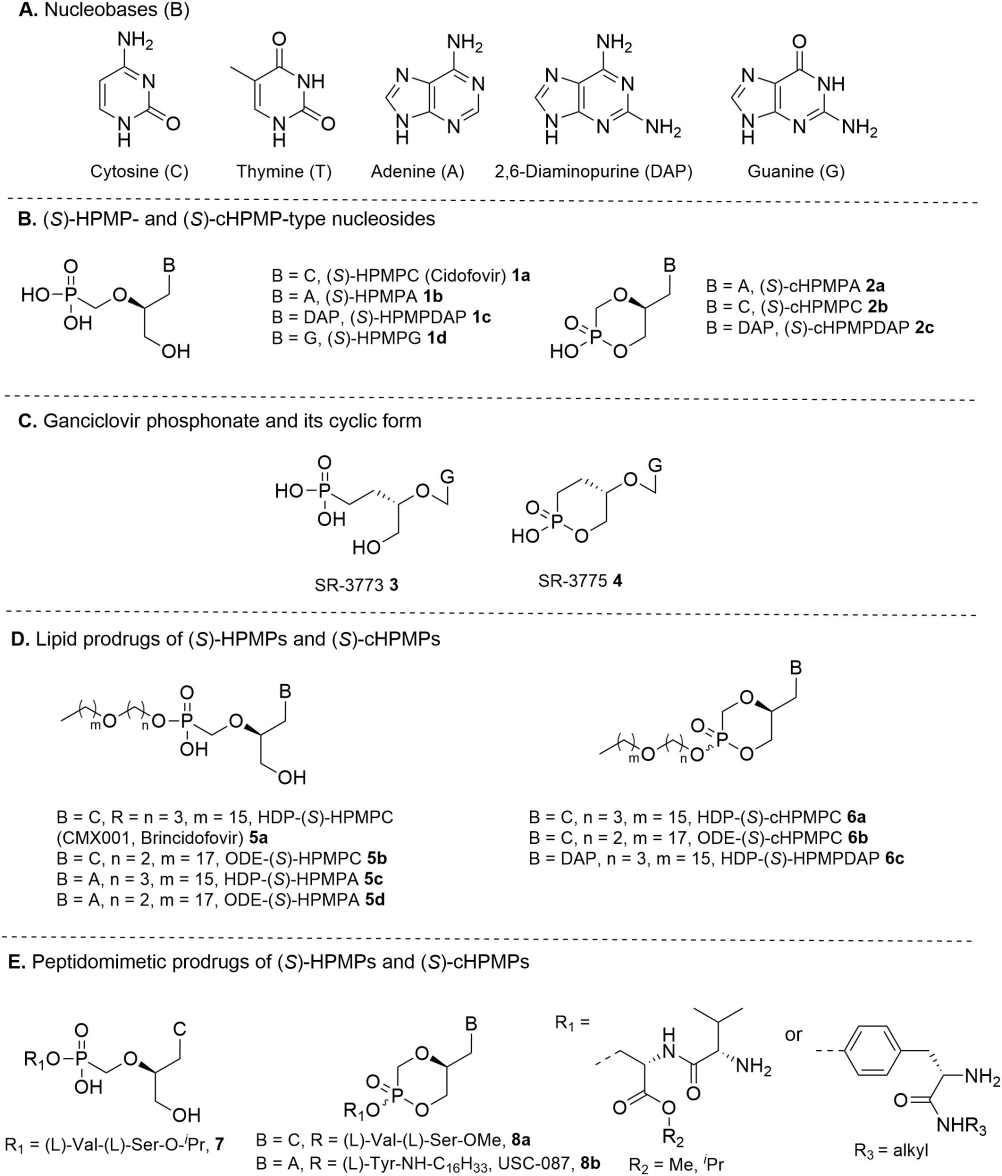
**(A)** Common pyrimidine and purine nucleobases found in nucleoside phosphonates; **(B)** (S)-HPMP and (S)-cHPMP-type nucleosides; **(C)** ganciclovir phosphonate and its cyclic form; **(D)** lipid prodrugs of (S)-HPMPs and (S)-cHPMPs; **(E)** peptidomimetic prodrugs of (S)-HPMPs and (S)-cHPMPs.

Herpesviruses (Herpesviridiae family) are ubiquitous, large double stranded DNA viruses responsible for a variety of latent and recurrent infections in specific tissues. Among the more than hundred recognized species, nine types of herpesviruses are known to routinely use humans as their primary host, including herpes simplex virus types 1 and 2 (HSV-1 and HSV-2), varicella-zoster virus (VZV), human cytomegalovirus (HCMV), human herpesviruses 6A, 6B, and 7 (HHV-6A, HHV-6B, and HHV7), as well as oncogenic Epstein-Barr virus (EBV) and human herpesvirus 8 (HHV8) or Kaposi's sarcoma-associated herpesvirus (KSHV). In addition, a herpesvirus carried by macaques known as B virus may also occasionally infect humans. Diseases caused by human herpesviruses pose a particularly significant risk of morbidity and mortality in severely immunocompromised individuals. An important breakthrough in herpesvirus therapy coincided with the development of nucleoside analogs bearing an acyclic pseudo-sugar moiety (e.g., acyclovir, ganciclovir, and penciclovir), which culminated in the late 1980's with the discovery of acyclic nucleoside phosphonates (ANPs) (de Clercq and Holy, 2005). ANPs are nucleotide analogs characterized by a non-hydrolysable P–C bond linking a phosphonate moiety to various aliphatic side chains of purine and pyrimidine acyclic nucleosides. Unlike “classical” anti-herpes nucleosides, the intracellular activation of ANPs is not dependent on a viral-encoded kinase to promote their initial phosphorylation. Generally, ANPs are divided in different subclasses depending on the chemical structure of their side chain residue, to whom corresponds a specific antiviral activity spectrum (de Clercq, 2007). In particular, the presence of a 2′-hydroxymethyl group as in the 1-(3-hydroxy-2-phosphonylmethoxypropyl) series (HPMPs, Figures 1A,B) led to broad-spectrum agents with potent and selective activity against a variety of herpesviruses as well as other double-stranded DNA viruses (pox-, polyoma-, papilloma-, and adenoviruses) (Declercq et al., 1986, 1987). The antiviral potency of HPMPs was found to be strongly influenced by the stereochemistry at the C2′ position with a preference for the (*S*)- over (*R*)-enantiomers. The cytosine analog (*S*)-HPMPC **1a** (cidofovir, Vistide®) is the flagship member of HPMPs (Declercq et al., 1987), and the first ANP to have been clinically approved in 1996 for the treatment of HCMV retinitis, the most common intraocular opportunistic infection affecting AIDS patients. It has also found “off-label” use for the systemic or topical treatment of herpesvirus infections resistant to first line therapy and other diseases, such as papillomatosis and progressive multifocal leukoencephalopathy due to JC polyomavirus. Moreover, (*S*)-HPMPC administered by parenteral injection has been shown to protect against lethal smallpox infection. Within the cell, (*S*)-HPMPC is converted by cellular kinases to its diphosphate metabolite (*S*)-HPMPCpp, which acts as a competitive substrate for incorporation into the viral genome by a DNA polymerase. (*S*)-HPMPCpp was found to possess a 25- to 50-fold greater affinity for viral over host polymerases, thus allowing for selective inhibition of DNA virus replication.

As compared to (*S*)-HPMPC, the adenine congener (S)-9-(3-hydroxy-2-phosphonylmethoxypropyl)adenine (*S*)-HPMPA **1a** also displayed a similarly potent *in vitro* inhibitory activity against a broad panel of DNA viruses (Declercq et al., 1986), with EC_50_ values in the 0.08–13 μM range for different herpesviruses (HSV-1, HSV-2, VZV, HCMV, EBV, and HHV-6), 0.8–0.17 μM for adenoviruses (Ad5 and Ad8), 2.3 μM for vaccinia virus, and 15 μM for human polyoma virus. (*S*)-HPMPA was also reported to inhibit human hepatis B virus (HBV) with an EC_50_ = 1.5 μM (Yokota et al., 1991). However, a less favorable safety profile emerged from *in vitro* toxicity studies that halted its progressing into clinical development (Andrei et al., 1991). Other purine containing derivatives, i.e., 2,6-diaminopurine and guanine congeners (*S*)-HPMDAP **1c** and (*S*)-HPMG **1d**, were also found to exert broad anti-DNA virus activity *in vitro*.

Studies in multiple animal models as well as clinical testing in humans evidenced nephrotoxicity as a clear dose-limiting side effect of (*S*)-HPMPC (Bischofberger et al., 1994; Lalezari et al., 1995, 1997, 1998), and a major obstacle to the clinical application of ANPs. Upon intravenous administration, the drug accumulates in the kidney with a concentration 100-fold higher than other tissues, leading to local cellular toxicity that manifests as elevated levels of serum creatinine as well as proteins in the urine. This adverse effect was explained by the interaction of (*S*)-HPMPC with renal organic anion transporter 1 (OAT1) and consequential higher transport rates at the level of the basolateral and/or luminal membranes of the proximal convoluted tubule (PCT) cells compared to the efflux of the drug at the luminal side (Ho et al., 2000).

In this regard, it was demonstrated how a particularly beneficial effect could be derived from the generation of cyclic phosphonoesters, which were simply obtained by an intramolecular esterification reaction between the primary free 3′-OH group and neighboring phosphonic acid moiety (Rosenberg and Holy, 1987; Bischofberger et al., 1994). These cyclic congeners of HPMPs, as exemplified by (*S*)-cHPMPA **2a** and (*S*)-cHPMPC **2b** (Figure 1B), were found to retain the pronounced anti-herpesvirus potency of their parent compounds by acting as their intracellular prodrugs, while allowing for greater selectivity indexes. It was demonstrated that (*S*)-cHPMPC underwent an efficient cCMP phosphodiesterase-mediated *in vivo* conversion to the parent phosphonate (Mendel et al., 1997), leading to accumulation of similar levels of the active metabolite (*S*)-HPMPCpp. On the other hand, several studies documented that (*S*)-cHPMPC administered to diverse animal species (e.g., rats, rabbits, and monkeys) contributed to a substantially lower nephrotoxicity than (*S*)-HPMPC due to the presence of a modified charge that made it less susceptible to the action of organic anion transporters at the level of PCT cells.

Owing to the success of HPMPs, the introduction of a phosphonate group into the side chain of acyclic nucleosides licensed for clinical use as anti-herpesvirus agents [e.g., acyclovir and ganciclovir (GSV)] was also investigated. The (*R*)-enantiomer of ganciclovir phosphonate **3** (Figure 1C) exhibited potent activity against HMCV both in cell culture and murine models (Smee et al., 1994), while its cyclic phosphonate analog **4** was associated with a substantially reduced toxicity as a result of a similar effect as that observed for cyclic HPMPs (Smee and Reist, 1996).

While the antiviral activities of all these compounds are indeed remarkable, their utility, especially in clinical practice, is mitigated by their poor oral bioavailability. It is not surprising that the presence of ionizable groups at physiological pH restricts their ability to penetrate the lipid-rich cellular membrane, thus requiring parental administration in order to be effective. During the past years, various solutions have been sought to develop orally active prodrug forms with enhanced absorption and pharmacological parameters, few of which have progressed to *in vivo* or clinical studies. Prodrug derivatization was performed both at the level of acyclic and cyclic HPMP nucleoside phosphonates by covalently linking a suitable promoiety via an ester or amide bond. Below we provide selected illustrative examples for both categories.

In a systematic comparative study with a set of alkoxyalkyl monoesters of various ANPs analogs, Hostetler et al. defined the design principles for generating lipid-HPMP conjugates that could be sufficiently hydrophobic to enable improved cellular uptake and absorption by mimicking natural cellular membrane phospholipids (Figure 1D). Side chain moieties up to 20–21 atoms long appeared to be necessary for achieving optimal antiviral activity combined with enhanced oral bioavailability. Several *in vitro* studies demonstrated that the most active analogs hexadecyloxypropyl (HDP)- and octadecyloxyethyl (ODE)-(*S*)-HPMPC **5a** and **5b** showed a similar multiple log increases in antiviral activities over their parent molecule against herpesviruses (e.g., up to 1,000-fold for HCMV) (Beadle et al., 2002; Williams-Aziz et al., 2005). Low nanomolar EC_50_ values were obtained against wild-type as well as GCV-resistant HCMV strains (EC_50_ = 0.9 nM), VZV (EC_50_ = 0.1–0.4 nM), HSV-1 (EC_50_ = 0.02–0.06 μM), HSV-2 (EC_50_ = 0.008–0.01 μM), HHV-8 (0.02–0.03 μM), and EBV (0.03–0.10 μM). In addition, alkoxyalkyl esters HDP-(*S*)-HPMPC and ODE-(*S*)-HPMPC exhibited >100-fold enhanced *in vitro* activity vs. the parent nucleoside against cells infected with orthopoxviruses (poxviruses), such as vaccinia virus (VV) and cowpox (CV) (Kern et al., 2002), adenovirus (Hartline et al., 2005), BK polyomavirus (BKPyV) (Randhawa et al., 2006), and papillomavirus. Further evidence for the versatility of this prodrug approach was provided with the synthesis of related prodrugs of (*S*)-HPMPA (compounds **5c** and **5d**) (Beadle et al., 2006; Lebeau et al., 2006), (*S*)-cHPMPC (compounds **6c** and **6d**) (Beadle et al., 2002; Kern et al., 2002), and (*S*)-cHPMPDAP (**6c**) (Krecmerova et al., 2010), which likewise allowed for a significant enhancement of bioavailability as well as antiviral potency against herpesviruses and poxviruses.

HDP-(*S*)-HPMPC **5a**, also known as CMX001 or brincidofovir, is the most well-investigated compound of this series, and the only orally available HPMP prodrug to have undergone clinical development so far (Chimerix Inc.). Originally intended as an oral formulation to treat smallpox in the event of a variola virus accidental outbreak or for biodefence purposes, it later underwent clinical trials for the treatment of HCMV and ADV infections. Brincidofovir remains intact in the plasma throughout the body and is absorbed in the small intestine by means of facilitated and passive diffusion processes. *In vitro* metabolic studies reported 10- to 20-fold increased membrane permeation rates and longer intracellular half-life (10 days) for brincidofovir when compared to (*S*)-HPMPC (2.7 days) (Aldern et al., 2003). Brincidofovir is converted intracellularly to (*S*)-HPMPCpp after cleavage of its lipid moiety and phosphorylation by intracellular kinases. Analysis of cellular metabolites showed that levels of (*S*)-HPMPCpp were 100-fold greater with brincidofovir than those observed with (*S*)-HPMPC.

Oral administration of brincidofovir proved as effective as parental (*S*)-HPMPC in the treatment of herpes- and poxvirus infection in several animal models, however, unlike (*S*)-HPMPC, brincidofovir was not a substrate of OAT1 and did not concentrate in renal proximal tubules, as shown by studies evaluating tissue distribution of radiolabeled species in mice (Ciesla et al., 2003).

Following promising Phase I safety and pharmacokinetic studies (Painter et al., 2012), brincidofovir was evaluated in a Phase II dose escalation trial in HCMV-seropositive recipients of hematopoietic-cell transplant (HCT), which identified 100 mg orally twice weekly as the maximum tolerated dosage to significantly reduce the incidence of HCMV (Marty et al., 2013). However, a subsequent phase III placebo-controlled trial in the same patient population showed that the incidence of clinically significant CMV infections and mortality was similar and higher, respectively, when comparing brincidofovir-treated patients and placebo recipients. Furthermore, serious adverse gastrointestinal events (digestive symptoms), such as acute graft-vs.-host disease (GVHD) and diarrhea were more frequently observed among treatment group participants (Marty et al., 2019).

Although clinical trials of brincidofovir for the prevention of HCMV are currently suspended, its evaluation for the treatment of disseminated adenovirus infections is ongoing and a phase II trial in pediatric and adult allogenic HSCT recipients was recently completed (Grimley et al., 2017) (ClinicalTrials.gov Identifier: NCT02087306; Recruitment Status: Completed). Oral administration of brincidofovir twice a week was reported to decrease mortality and led to an early and marked decline in HAdV viremia in subjects with HAdV levels of >1,000 genome copies/mL at baseline, although the results were not statistically significant.

An additional approach that was devised to counter the challenges associated with the oral delivery of HPMPs include the use of non-toxic biomoieties, such as single amino acids or peptides, with the aim of further facilitate gastrointestinal drug absorption by transporter-mediated uptake (Figure 1E). By linking a P-OH group of either (*S*)-HPMPC **1a** or (*S*)-cHPMPC **2c** to various dipeptides via the side chain hydroxy group of a (L)-serine alkyl ester residue, Mckenna et al. prepared a series of peptidomimetic conjugates that inhibited HCMV replication in the submicromolar range, while exhibiting enhanced permeability and bioavailability relative to the parent molecules (McKenna et al., 2005; Eriksson et al., 2008; Peterson et al., 2011). In a murine model, oral administration of (L)-Val-(L)-Ser prodrug conjugates **7** and **8a** led to a 15- and 8-fold enhancement in plasma levels of total (*S*)-HPMPC species, respectively, over those obtained from dosing with the parent drug (Eriksson et al., 2008; Nadel et al., 2014). While the uptake mechanism underpinning the improved absorption of these prodrugs might rely on the function of specific transporters located in the gastrointestinal tract, further studies demonstrated that these compounds were not substrates of the peptide-specific intestinal transporter human hPEPT1 (hPEPT1) (Peterson et al., 2010).

Another proof of principle was achieved with a series of *N*-alkyl tyrosinamide ester prodrugs of (*S*)-cHPMPC and (*S*)-cHPMPA (Zalcharova et al., 2011). These derivatives underwent a simple activation pathway by generating the parent cyclic form as well as an acyclic form bearing the respective prodrug moiety, which was also endowed with *in vitro* antiviral activity. This prodrug design afforded analogs with a comparable *in vitro* inhibitory potency as the corresponding parent molecules against HCMV, HSV-1, VV, and CV, corresponding to IC_50_ values ranging from 0.1 to 50 μM, while enabling superior oral bioavailability *in vivo*.

The most promising example of this series described so far consists of an *N*-hexadecyl tyrosinamide congener of (*S*)-cHPMPA identified as USC-087 (**8b**), whose efficacy against various human adenovirus types was demonstrated in cell culture and *in vivo*. In the immunosuppressed Syrian hamster model, oral administration of USC-087 completely prevented or significantly decreased mortality against HAdV infection up to 4 days post-lethal intravenous challenge. Owing to the ability to decrease virus burden and liver damage, protection of the animal was achieved even when the administration of **8b** started 4 days after challenge (Toth et al., 2018).

**Figure 2:**
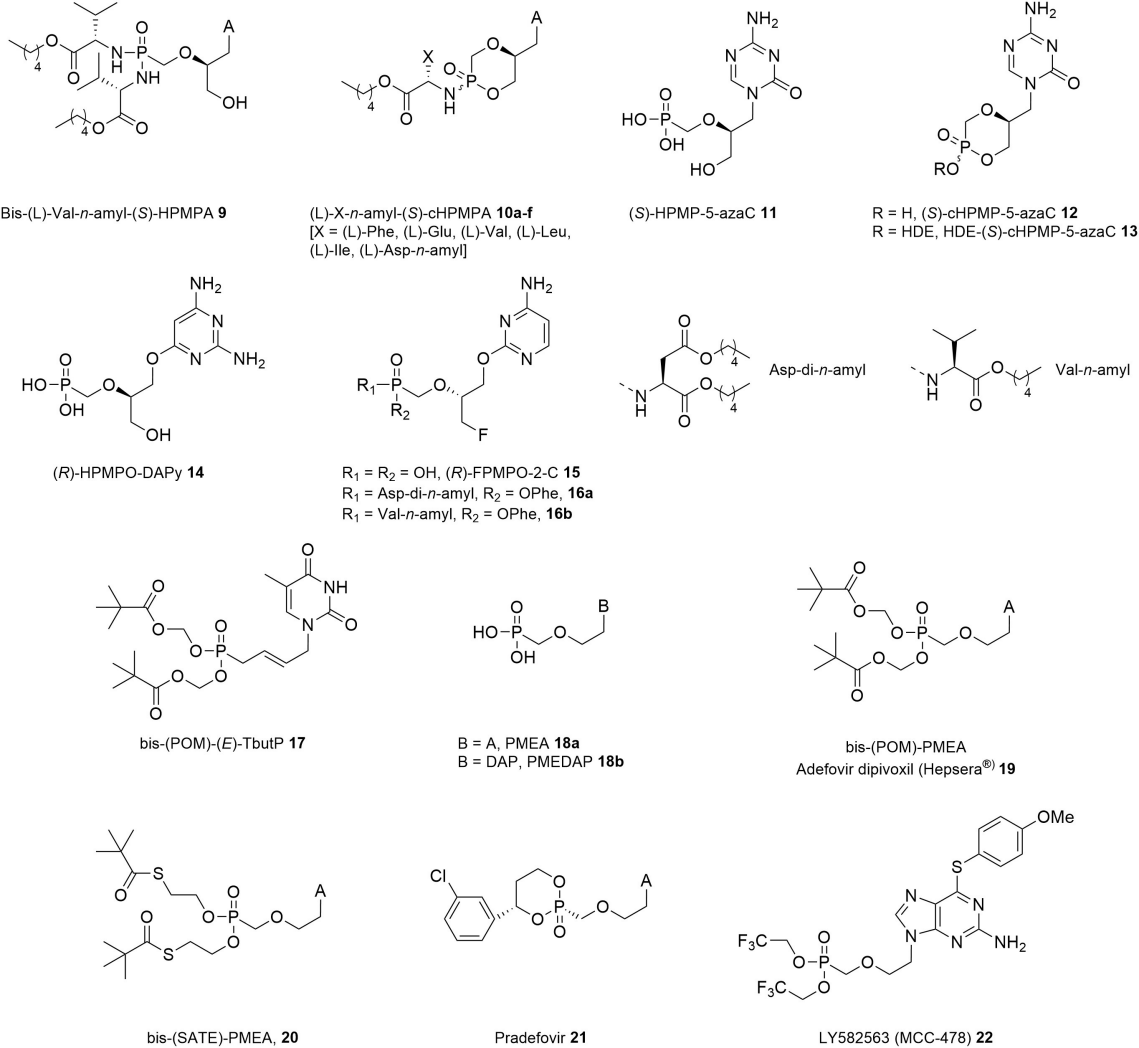
Phosphonamidate prodrugs of (S)-HPMPA and (S)-cHPMPA, (S)-HPMP-5-aza C and its prodrugs, O-6 and O-2-alkylated HPMP analogs, an alkenyl based bis-(POM) ANP prodrug, PME-type nucleosides and prodrugs.

A more recent application of the prodrug strategy to HPMPs can be found in the synthesis of phosphonobisamidate (**9**) and aryloxyphosphonamidate analogs (**10a–f**) of (*S*)-HPMPA and (*S*)-cHPMPA, respectively (Figure 2) (Luo et al., 2018). Overall, these compounds displayed a dramatic enhancement of antiviral activity compared to the reference drug (*S*)-HPMPC when tested against several herpesviruses including HSV-1, HSV-2, VZV, and HCMV, which was explained by their increased lipophilicity (as expressed by their clogP values). Within the (*S*)-cHPMPA *n*-amyl ester amidate series, the EC_50_ values varied depending on the amino acid moiety but largely persisted in the low nanomolar range. Selectivity indexes up to 20,000 and 1,800 against VZV and HCMV, respectively, were obtained. Furthermore, these prodrugs were found to be stable in human plasma with *t*_1/2_ values exceeding 1 h, while undergoing fast metabolism in human liver microsomes.

The first generation HPMPs was promptly complemented by other ANPs combining a similar acyclic motif with non-natural pyrimidine nucleobases, which showed comparable antiviral activity in cell culture and animal models as the first generation HPMPs, but have not yet been assessed for their clinical potential. One such compound that deserves a special mentioning is the *sym*-triazine isomeric analog of (*S*)-HPMPC, namely (*S*)-HPMP-5-azaC (**11**, Figure 2), which displayed a broad anti-DNA virus spectrum closely resembling that of its parent drug with equipotent inhibitory activity against HSV-1 (EC_50_ = 0.07 μg/mL), HSV-2 (EC_50_ = 0.25 μg/mL), and vaccinia virus (EC_50_ = 2.56 μg/mL), while being 2- to 7-fold more active against VZV, HCMV, HHV-6, and Ad2 (EC_50_ = 0.02–0.71 μg/mL) (Krecmerova et al., 2007a). In contrast, both its (*R*)-enantiomer and isomeric 6-azacytosine analogs were significantly less active. In addition, (*S*)-HPMP-5-azaC also proved to be effective inhibiting Moloney murine sarcoma virus (MSV)-induced transformation of C3H/3T3 cell cultures (EC_50_ = 2.2 μg/mL).

In analogy to previous work on HPMPs, the corresponding cyclic form (*S*)-cHPMP-5-azaC **12** revealed strong *in vitro* activity against DNA viruses, with EC_50_ values in the range of 0.06–3.1 μg/mL, comparable with those of (*S*)-HPMP-5-azaC, (*S*)-HPMPC, and (*S*)-cHPMPC. Further derivatization of (*S*)-cHPMP-5-azaC with a lipid ester moiety led to a general enhancement of antiviral activity, with the most potent hexadecyloxyethyl (HDE) prodrug **13** exhibiting a 58- to 250-fold increased activity (Krecmerova et al., 2007b). As compared to (*S*)-HPMPC, (*S*)-HPMP-5-azaC displayed a 2- to 16-fold higher selectivity index against adeno-, pox, and herpesviruses in cell culture. However, despite their favorable toxicity profile, neither 5-aza-(S)-HPMPC nor its HDE prodrug were further pursued for their therapeutic potential, mainly due to the instability and complex metabolic pathways involving chemical decomposition and enzymatic deamination.

Another interesting compound was discovered by linking the HPMP pseudosugar synthon through an oxygen atom to the C6 of 2,4-diaminopyrimidine (DAPy) in place of the classical N1-pyrimidine linkage (Figure 2). The resulting (*R*)-HPMPO-DAPy **14** inhibited HSV-1, HSV-2, and VZV replication at a level comparable to (*S*)-HPMPA (**1b**) with EC_50_ values in the 0.51–51 μM range, while being a poor inhibitor of HCMV (EC_50_ = 139 μM vs. 0.63 μM of **1b**) (Balzarini et al., 2004). (*R*)-HPMPO-DAPy proved also to be active in cell culture against other DNA viruses, including adenovirus type 2 (EC_50_ = 5.4–10 μM), albeit to a 3- to 10-fold lesser extent than the parent compounds (*S*)-HPMPA and (*S*)-HPMPC (Naesens et al., 2005), and especially poxviruses, e.g., camel pox, cowpox, vaccinia, and orf virus (Balzarini et al., 2004; Dal Pozzo et al., 2005; de Clercq et al., 2005; Duraffour et al., 2007). Promising results in animal models mimicking disseminated vaccinia further emphasized the antiviral potential of **14** in rivaling the utility of (*S*)-HPMPC (**1a**) for the treatment of human poxvirus infections and suppression of vaccine-related complications following immunization of immunocompromised hosts (de Clercq and Neyts, 2004; Stittelaar et al., 2006).

Recently, Herdewijn et al. described a new family of *O*-alkylated ANP amidate prodrugs with potent activity against different strains of HMCV and VZV (Figure 2) (Luo et al., 2020). The main difference with the previous series is that the aliphatic side chain is linked to the 2-*O*-position of a pyrimidine base rather than the 6-*O* position. Among the parent phosphonates, (*R*)-*O*-2-alkylated 3-fluoro-2-(phosphonomethoxy)propyl cytosine (FPMPC) **15** was found to be a moderately active inhibitor of HCMV and VZV with EC_50_ values in the 0.80–2.36 and 3.79–25.3 μM range, respectively. Interestingly, subsequent derivatization of (*R*)-O-2-alkylated FPMPC as its L-aspartic acid or L-valine containing phenoxyamidate prodrugs **16a** and **16b** led to a marked improvement of activity with EC_50_ values ranging between 0.094 and 0.54 μM along with low cellular toxicity. In addition, it should be noted that early attempts to tune the activity of HPMPs through replacement of the 3′-hydroxyl group by a fluorine atom resulted in loss of antiviral activity against herpesviruses (Balzarini et al., 1993; Luo et al., 2017). However, diamyl aspartate phosphonoamidate prodrugs of purine containing *N*-alkylated FPMP analogs exhibited submicromolar anti-VZV, while (*S*)-aspartate-FPMPC was endowed with an EC_50_ value of 0.76 μM against HCMV (Luo et al., 2017).

A series of 5-substituted pyrimidine ANPs bearing an unsaturated acyclic side chain was also synthesized along with their bis-pivaloyloxymethyl (POM) ester prodrugs (Topalis et al., 2011). Among them, bis-POM-(*E*)-thymidine-but-2-enyl phosphonate **17** was found to be the most potent inhibitor of HSV-1 and HSV-2 with IC_50_ in the 3 and 6 μM range, respectively, as well as VZV (IC_50_ = 0.19 μM), while the corresponding (*Z*)-configured congener was completely devoid of anti-DNA virus activity. It was argued that the observed *in vitro* antiviral activity correlated with the ability of **17** to act as a substrate for human thymidine monophosphate (TMP) kinase.

## Anti-Hepadnaviral and Anti-Retroviral Nucleoside Phosphonates

According to the latest WHO estimates, about 38 million people worldwide are living with HIV (2019 WHO report), while chronic HBV infection accounts for a global prevalence of 257–291 million affected individuals (2015 WHO Global hepatitis report and 2016 Polaris Observatory study). Due to the highly contagious capacity and common transmission routes shared by these bloodborne viruses, high rates (5–20%) of HIV-HBV co-infection are frequent, which may exacerbate clinical complications, such as the progression of the HBV infection to liver cirrhosis and hepatocellular carcinoma. Moreover, antiviral drug resistance may often develop after prolonged treatments usually resulting in progressive loss of clinical benefit.

The evolution of ANP inhibitors occurred in parallel with structural simplifications at the acyclic chain and developed into an important achievement with regard to their activity spectrum. By extending their seminal studies on ANPs, Holy, and De Clercq conducted similar investigations into the SAR profile of related 2-phosphonylmethoxyethyl (PME) derivatives (Figure 2). In striking contrast to the HPMP series, PME nucleosides lacking chirality due to the absence of the 2′-substituent exerted a distinctive 2-fold inhibitory effect against both DNA viruses of the herpes (Declercq et al., 1986, 1987) and hepadnavirus (Yokota et al., 1991; Heijtink et al., 1994) families as well as retroviruses of different species (Pauwels et al., 1988; Naesens et al., 1997). On the other hand, no appreciable activity was observed against adeno-, pox-, and papillomaviruses. Furthermore, only purine containing PMEs exhibited antiviral activity that comprise the most successful member of this class, i.e., PMEA or adefovir (**18a**, Figure 2) (Declercq et al., 1986). This adenine congener is arguably one of the most interesting ANPs due to its broad activity spectrum, and was selected as candidate for further clinical development especially in view of its potent reverse transcriptase inhibitory activity in various subtypes of HIV-1 clinical isolates and several animal models. While exhibiting a 5-fold increased antiretroviral (MSV) efficacy than **18a**, the diaminopurine containing congener PMEDAP **18a** (Figure 2), displayed an inferior toxicity profile (Naesens et al., 1989).

These initial findings motivated the generation of an entire family of phosphonodiester prodrugs of PMEA, as a means for oral delivery (Figure 2). Eventually, adefovir came to the market in 2002 as its acyloxy ester prodrug adefovir dipivoxil [bis-(POM)-PMEA, ADV, Hepsera®, **19**] (Starrett et al., 1992; Cundy et al., 1994). Although initially evaluated for the treatment of HIV-infected individuals, the compound proved too nephrotoxic at the indicated efficacy dose (125 mg-dose once daily) to permit long term use (Kahn et al., 1999). It was nonetheless approved for oral chronic anti-HBV therapy as in this case administration at submaximally efficacious doses (10 mg-dose once daily) was sufficient to achieve an effective inhibitory activity (Hadziyannis et al., 2003; Marcellin et al., 2003; Peters et al., 2004).

Complementary approaches to the bis-(POM) prodrug strategy were considered in order to minimize systemic distribution and especially kidney exposure to PMEA and its metabolites, while enhancing liver targeting delivery.

Symmetrical phosphonodiester prodrugs of PMEA bearing carboxyesterase-labile S-acyl-thioethyl (SATE) masking moieties, as exemplified by bis(*t*-Bu-SATE)-PMEA **20** (Figure 2), exhibited antiviral potencies against HIV-1 similar to bis-(POM)-PMEA (EC_50_ = 0.03–0.65 μM for **20** vs. 0.04–0.08 μM for **19**), while being characterized by a greater stability in human gastric juice and human serum (Benzaria et al., 1996). However, further clinical development of this class of prodrugs was precluded by concerns associated with the mutagenic potential of their degradation byproducts.

A remarkably pronounced enhancement of plasma stability compared to **19** was observed with the selectively liver-activated cyclic 1-aryl-1,3-propanyl ester prodrug of PMEA (**21**, Figure 2), also known as pradefovir (MB-06866) (Lin et al., 2006). The design of **21** was based on the HepDirect™ prodrug technology, which relied on the oxidative conversion of **21** to PMEA by the action of the hepatic cytochrome P450 isozyme CYP3A4, consequently allowing for decreased non-hepatic activation. Preclinical evaluation of pradefovir in rats showed a 12-fold improvement in the liver/kidney ratio for **21** over **19** combined with a good oral bioavailability (42%) as a mesylate salt formulation (Reddy et al., 2008). A 48-weeks Phase II trial involving chronically infected HBV patients further confirmed that pradefovir mesylate (dosed at 5–30 mg/day) was well-tolerated and significantly more active than **19** (dosed at 10 mg/day), without producing significant changes in kidney function markers (Lee et al., 2006). Although further clinical evaluation of **21** was discontinued in USA and Europe due to the potential carcinogenic effect observed in animal studies, a Phase III trial for the treatment of patients with chronic HBV infections is currently ongoing in China to determine (ClinicalTrials.gov Identifier: NCT04543565; Recruitment Status: Recruiting) (Zhang et al., 2020).

In contrast to the broad spectrum of activity characteristic of PMEs bearing natural nucleobases, bis(trifluoroethyl) esters of 2-amino-6-arylthio 9-[2-(phosphonomethoxy)ethyl]purines, and particularly 6-(methoxyphenyl)thio substituted derivative **22** (LY582563, Figure 2), were found to possess potent anti-HBV antiviral activity *in vitro* against wild-type and lamivudine resistant variants, while lacking inhibitory activity against HIV-1 (Ono-Nita et al., 2002; Sekiya et al., 2002). Compound **22** was absorbed in the gastrointestinal tract when administered orally to mice and high concentrations of its main active metabolite were observed in the liver (Sekiya et al., 2002). Its clinical efficacy for the treatment of HBV-infected patients was demonstrated in phase I studies (Wise et al., 2002).

**Figure 3:**
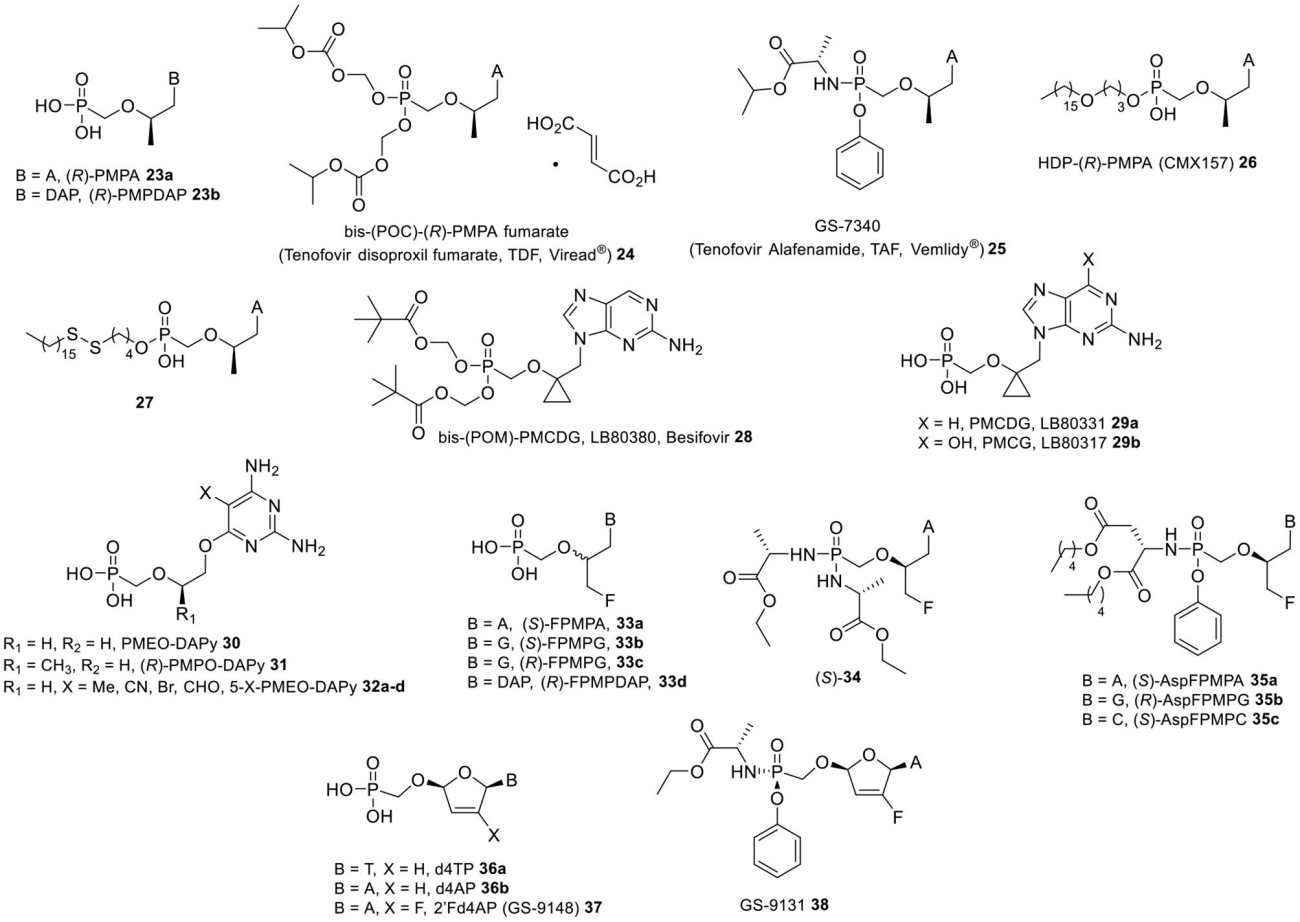
PMP-type nucleosides and prodrugs, Besifovir and its metabolites, PMEO- and PMPO-DAPy-type nucleosides, FPMP-type nucleosides and prodrugs, 2′, 3′-didehydro-2′,3′-dideoxyribose nucleoside phosphonates, and prodrugs.

The presence of a methyl group at the 2′-position of the side-chain of ANPs define an additional series of derivatives endowed by a yet diverse antiviral activity spectrum, with the most notorious example being the (*R*)-enantiomer of 9-(2-phosphonylmethoxypropyl)adenine [(*R*)-PMPA] (**23a**, Figure 3), also known as tenofovir (Balzarini et al., 1993). PMPs with a purine nucleobase displayed pronounced antiretroviral activity in cell culture, e.g., against HIV-1, HIV-2, and MSV, while completely lacking anti-herpesvirus activity. These analogs also resulted potent inhibitors of hepadnaviruses including human and duck HBV (Heijtink et al., 1994). Although in these early studies the 2,6-diaminopurine congener (*R*)-PMPDAP **23b** was found to be up to 35- and 8-fold more potent than (*R*)-PMPA in inhibiting HIV and HBV replication, respectively, it was (*R*)-PMPA that later entered and underwent successful clinical development. In order to enable oral administration, (*R*)-PMPA is currently marketed as its bis(isopropyloxycarbonyloxymethyl) ester prodrug tenofovir disoproxil fumarate (**24**) (Naesens et al., 1998; Robbins et al., 1998) for the treatment of HIV and chronic HBV infections (Fung et al., 2002; Kuo et al., 2004; Heathcote et al., 2011), either as single drug (TDF, Viread®) or in a fixed-dose combination with other antiretroviral agents, such as emtricitabine (Truvada®) and efavirenz (Atripla®). TDF offered clear advantages in terms of bioavailability of the active drug (25 vs. 2% the dianionic parent compound **23a**) upon oral administration to mice, as well as good tissue distribution and biological stability (Kearney et al., 2004). However, chronic treatment with TDF has been linked to lactic acidosis, Fanconi syndrome, acute renal failure, and bone loss mainly due to its cleavage in the systemic circulation (Duarte-Rojo and Heathcote, 2010). Therefore, alternative prodrugs strategies have been explored to decrease plasma exposure to (*R*)-PMPA.

For instance, the alanyl phenyl ester phophonoamidate prodrug of (*R*)-PMPA, namely tenofovir alafenamide **25** (TAF), was specifically designed to target lymphatic cells and tissue, where it was shown to undergo fast cleavage mediated by a lysosomal carboxypeptidase cathepsin A, while being endowed with higher plasma stability than **24** as well as little or no nephrotoxicity (Lee et al., 2005; Birkus et al., 2007). Notably, TAF was found to be 1,000-fold more active against HIV-1 (EC_50_ = 5 nM) when compared to the parent nucleoside **23a** (EC_50_ = 5 μM), and was also licensed for clinical use in adults infected with chronic HBV (Ray et al., 2016).

Lipid conjugate ester prodrugs of (*R*)-PMPA have also been shown to provide a valuable mean for facilitating tissue targeted delivery, while minimizing systemic exposure to the parent drug. For instance, HDP-(*R*)-PMPA **26** (CMX-157, tenofovir exalidex TXL™) was shown to remain intact in the bloodstream, while undergoing cleavage by an intracellular hydrolase (phospholipase C and/or sphingomylenase), to liberate (*R*)-PMPA within the cytosol. This prodrug was reported to have 267- and 4.5-fold more pronounced *in vitro* activity than (*R*)-PMPA against HIV-1 and HBV, respectively (Painter et al., 2007), and could effectively suppress the replication of nucleos(t)de reverse transcriptase inhibitor (NRTI)-resistant HIV variant strains with low nanomolar EC_50_ values (Lanier et al., 2010). It showed good oral bioavailability *in vivo*, while being well-tolerated at doses up to 200 mg/kg in rats and monkeys with gastrointestinal toxicity being dose-limiting (Lanier et al., 2009). In early phase clinical trials, **26** showed a favorable tolerability and good pharmacokinetic properties, retaining significant antiviral activity for up to a week after a single administration. A multiple ascending dose proof of concept Phase II study involving HBV infected subjects treated orally with CMX157 at increasing dose levels up to 100 mg to evaluate the safety and tolerability was completed (ClinicalTrials.gov Identifier: NCT02710604; Recruitment Status: Completed) (Tanwandee et al., 2017).

Liotta et al. recently developed disulfide-based lipid conjugates of (*R*)-PMPA that exhibited a 600-fold improved potency over the parent nucleoside phosphonate against HIV-1 and significantly higher therapeutic index of >100,000 (vs. 9,500 of TDF) for the most potent conjugate bearing a C16 lipid chain along with a S–S moiety five atoms away from the phosphonate (compound **27**). These conjugates were also potent inhibitors of HBV replication in hepatocytes with EC_50_ values comparable to TDF, and were stable in human plasma for >24 h (Giesler and Liotta, 2016; Giesler et al., 2016). Although it was demonstrated that these prodrugs had the potential to undergo intramolecular cleavage upon disulfide reduction, the principal mechanism accountable for the release of the parent drug and consequent *in vitro* antiviral activity seemed to be enzymatic hydrolysis, most likely mediated by the same enzymes involved in the decomposition of CMX-157 and other lipid conjugates.

In analogy to (*R*)-HPMPO-DAPy **14** (Figure 2), *O*-6-alkylated 2,4-diaminopyrimidine ANPs bearing either a PME or PMP aliphatic chain, i.e., PMEO-DAPy (**30**) and (*R*)-PMPO-DAPy (**31**) (Figure 3), were also synthesized and reported to be equipotent to their parent drugs [PMEA (**18a**) and (*R*)-PMPA (**23a**), respectively] in cells infected with HIV-1, HIV-2, and HBV (both wild-type and lamivudine-resistant strains), while being relatively non-toxic (Balzarini et al., 2002; de Clercq et al., 2005; Ying et al., 2005). Moreover, compounds **30** and **31** also inhibited MSV-induced cell transformation both *in vitro* (EC_50_ = 0.05–0.15 μM) and in a murine infection model (Balzarini et al., 2002). PMEO-DAPy derivatives featuring various substituents (e.g., Me, CN, Br, and CHO) at the 5-position of the DAPy base (compounds **32a-d**, Figure 3) were also shown to be potent inhibitors of HIV and HBV replication (Hockova et al., 2003; Ying et al., 2005). Notably, the 5-methyl PMEO-DAPy derivative **32a** was ~3- to 10-fold more active than PMEO-DAPy **30** against HIV and MSV including drug-resistant clinical isolates of HIV-1, while being more effective than PMEA in inhibiting retroviruses *in vivo* (Balzarini et al., 2007). The antiviral activity spectrum of PMEO-DAPy **30** was explained by the base pairing ability of the DAPy nucleobase, which was revealed to mimic the natural purine adenine counterpart allowing for hydrogen pairing to a pyrimidine nucleobase (Herman et al., 2010). In particular, PMEO-DAPy-pp was shown to be incorporate by HIV RT opposite to thymine (in DNA) or uracil (in RNA).

Since minor modifications at the side-chain of ANPs appeared to have a substantial impact on their antiviral activity spectrum, many different structural motifs have been explored for further antiviral profiling. Besifovir (LB80380, **28**, Figure 3), an orally available ANP dipivoxyl maleate prodrug featuring a cyclopropyl moiety at the 2′-position, is a clinical development candidate for the treatment of HBV (Choi et al., 2004; Papatheodoridis and Manolakopoulos, 2006). Besifovir was shown to undergo rapid deacetylation in the liver and intestine to its parent 2′-9-(2′-phosphonomethoxycyclopropyl)deoxyguanine **29a** (PMCDG, LB80331), which was further metabolized by enzyme oxidases (e.g., aldehyde and/or xanthine oxidase) to its corresponding guanine analog **29b** (PMCG) (Choi et al., 2004; Yuen et al., 2009). The diphosphate derivative of **29b** represents the active metabolite inhibiting viral DNA replication and eventually exerting the antiviral effect (Yuen et al., 2009). Besifovir was shown to exhibit high efficacy against both wild-type and HBV mutants resistant to lamivudine, adefovir, entecavir, and telbivudine studies as well as in animal models of HBV infection (Fung et al., 2008).

Results from various phase II trials indicated that **28** could significantly reduce the viral load in HBV-infected patients treated with escalating doses up to 240 mg/day for 4 weeks (Yuen et al., 2006), while also effectively suppressing HBV replication in treatment-naïve (Lai et al., 2014) and lamivudine-resistant chronic HBV patients (Yuen et al., 2010). Besifovir was also found to be clinically effective during a 2-years trial in. Although being generally well-tolerated and showing no significant nephrotoxicity, a high percentage of patients suffered from depletion of carnitine, suggesting that long-term use of besifovir needs to be accompanied by a carnitine supplement (Yuen et al., 2015). Recently, a phase III study demonstrated that **28** (150 mg) exerted antiviral efficacy comparable to TDF (300 mg) after 48 weeks of treatment with durable effects up to 96 weeks, together with comparably reduced bone and kidney toxicities (Ahn et al., 2019). These results led to the approval of **28** in South Korea (Besivo®).

As discussed in the previous section, 3-fluoro-2-(phosphonomethoxy)propyl (FPMP) nucleosides are side-chain fluorinated ANPs largely devoid of antiviral activity against a broad range of DNA viruses (Balzarini et al., 1993). As an exception, purine containing FPMP analogs (**33a–d**, Figure 3) were found to be effective inhibitors of HBV DNA synthesis (Heijtink et al., 1994; Luo et al., 2017), besides showing moderate antiretroviral activity against HIV-1 and HIV-2 in various cell lines (Balzarini et al., 1993; Luo et al., 2017). Notably, (*S*)-FPMPG **33b** was at least as potent as its (*R*) counterpart **33c** in inhibiting both HIV and HBV replication with the lowest EC_50_ values within this series (0.28–6.65 and 0.49–0.59 μM, respectively). On the other hand, the (*S*) enantiomer of FPMPA **33a** was 5- to 7-fold more active than (*R*)-FPMPA with EC_50_ values in the 3–6 and 1 μM range for HIV and HBV, respectively. The diaminopurine containing congener (*R*)-FPMPDAP **33d** was also endowed with antiretroviral activity (EC_50_ = 4.3 and 4.6 μM against HIV-1 and HIV-2, respectively) (Balzarini et al., 1993).

In an early report describing the application of the prodrug approach to FPMPs, a bis(amino acid) phosphonamidate of (*S*)-FPMPA (**34**) was synthesized that resulted in a 13-fold improved anti-HIV activity (EC_50_ = 0.54 μM) compared to the parent phosphonate (EC_50_ = 7.04 μM) (Jansa et al., 2011). Recently, a more systematic study revealed how the use of a diamyl aspartate phenoxyamidate motif to mask the phosphonate acid functionality of FPMPs resulted in a drastically enhanced antiretroviral potency by a factor up to 1,500, depending on the nucleobase moiety (Luo et al., 2017). Additionally, up to 160-fold improvements in anti-HBV activity were also observed, while some members of this family of prodrugs also displayed a broader spectrum of activity compared to their parent molecules by inhibiting herpesvirus replication. The (*S*)-FPMPA amidate prodrug **35a** emerged as the most active compound of this series, exhibiting anti-HIV-1 activity at low nanomolar concentrations in different cell lines along with excellent potency against HBV (EC_50_ = 0.01 μM) and VZV (EC_50_ = 0.05 μM). This prodrug was found to be stable in acid and human plasma media, while being efficiently metabolized in human liver microsomes with a half-life of 2 min. The (*R*) isomeric guanine derivative (R)-AspFPMPG **35b** also appeared to be a selective HIV, HBV, and VZV inhibitor, while being non-toxic to human hepatoblastoma cells. Notably, although a weak or no antiviral activity was generally associated with FPMP pyrimidine derivatives, the (*S*)-aspartate prodrug of FPMPC (**35c**) demonstrated anti-HCMV activity with an EC_50_ value up to 0.76 μM. Further studies are ongoing to determine the clinical potential of these compounds.

Numerous attempts have been made to identify cyclic nucleoside phosphonate (CNP) candidate antivirals; however, only a few of them have demonstrated to be effective HIV and HBV inhibitors with a promising therapeutic potential. Regardless of their closer structural resemblance to natural nucleoside monophosphates, CNPs mostly appear to be poor substrates for both viral and cellular kinases as well as being inefficiently incorporated by target polymerases. Extensive synthetic efforts on ribose-modified analogs helped to assemble important information about the SAR of these compounds (Mackman and Cihlar, 2011), revealing basic structural and electronic requirements for a favorable binding interaction with replicating target enzymes. For instance, the presence of a oxygen atom at the β-position to the phosphorus and a pKa value of the phosphonic acid functionality comparable to that of a phosphate moiety seemed to be fundamental prerequisites for potent antiretroviral activity. This is evident from thymine 2′,3′-didehydro-2′,3′-dideoxyribose nucleoside phosphonate **36a** (d4TP) and especially its adenine congener **36b** (d4AP) (Figure 1A and Figure 3), which were reported to exert a potent inhibitory effect on the replication of retroviruses including HIV-1 (Kim et al., 1991). Since d4AP was associated with mitochondrial toxicity, a fluorine atom was introduced at the 2′ position of the unsaturated ring moiety in order to increase the selectivity for HIV-1 reverse transcriptase (RT), while reducing the extent of the interaction with mitochondrial DNA polymerase γ. Although the resulting 2′Fd4AP (**37**, GS-9148) largely overcame this limitation showing more favorable pharmacological parameters than d4AP (Cihlar et al., 2008), further prodrug conjugation was needed for enhancing tissue specific delivery. Specifically, mono(ethyl-L-alanine) phosphonoamidate prodrug GS-9131 (**38**) did not lead to an improvement in the *in vitro* antiviral activity of **37**; however, about 20-fold high intracellular levels of the diphosphate metabolite of **37** were detected in PBMCs after oral administration of **38** to dogs (Cihlar et al., 2008; Ray et al., 2008). Following these observations, GS-9131 progressed into clinical evaluation for the treatment of patients infected with NRTI-resistant HIV-1, but failed to meet the targeted antiviral response in a Phase II trial (ClinicalTrials.gov Identifier: NCT03472326; Recruitment Status: Terminated).

**Figure 4:**
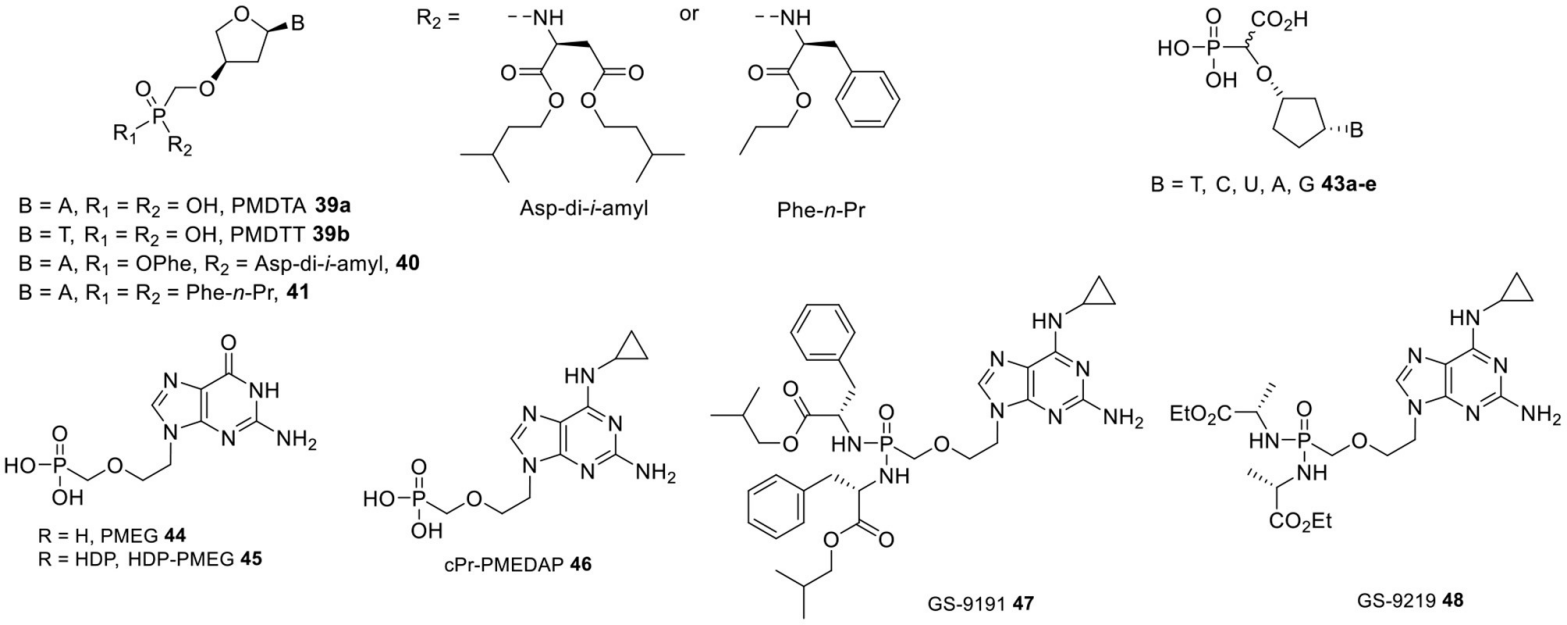
PMDT-type nucleosides and prodrugs, cyclopentyl α-carboxynucleoside phosphonates, PMEG and its prodrugs.

Another series of CNPs that constitute attractive clinical development candidates with a dual inhibitory activity against HIV and HBV are α-L-2′-deoxythreose nucleoside phosphonate analogs, in which a phosphonomethoxy group was bound at the 3′-position of a four-carbon sugar moiety (Figure 4) (Wu et al., 2005; Liu et al., 2016). This study further highlighted the decisive role played by the spatial proximity between the α-phosphorus atom and nucleobase, as comparatively greater bond distances, e.g., in modified five-carbon sugar CNPs bearing a phosphonate group at the primary hydroxyl group, appeared to be detrimental for antiviral activity. *In vitro*, phosphonomethoxydeoxythreosyl adenine (PMDTA) **39a** displayed low-micromolar EC_50_ values against HIV-1 and HIV-2 (4.69 and 5.23 μM, respectively) without affecting normal cell proliferation. The corresponding thymidine containing congener PMDTT **39b** was slightly less active with EC_50_ values in the 20 μM range. Likewise, PMDTA showed potent anti-HBV activity with an EC_50_ value of 0.5 μM, while PMDTT was 10-fold less potent (EC_50_ = 40.2 μM).

The use of the prodrug strategy was absolutely critical to boost the antiviral potency of these analogs. A variety of phosphonomonoamidate and phosphonodiamidate prodrugs were synthesized that showed a 100–1,000 increase of anti-HIV potency depending on the prodrug moiety, while 50- to 150-fold enhancements in activity against HBV were observed. The most potent congeners of this series were the L-aspartic acid diisoamyl ester phenoxy and L-phenylalanine propyl ester phosphonobisamidate prodrug of PMDTA **40** and **41** that exhibited anti-HIV and anti-HBV activities in the low nanomolar range (EC_50_ values up to 0.002 and 0.01 μM, respectively) and selectivity indexes of more than 300. These prodrugs were found to be reasonably stable at pH 8, while undergoing fast metabolic activation to the parent PMDTA in the presence of a carboxypeptidase Y type enzyme and human liver enzymes (Liu et al., 2016).

As mentioned above, the generally accepted intracellular metabolic cascade responsible for the antiviral effects of the majority of nucleoside phosphonates consists of two consecutive phosphorylation steps followed by interaction of the resulting diphosphate metabolite with a target replication enzyme and incorporation into viral DNA, which eventually induce absolute viral DNA chain termination. In contrast, alpha-carboxynucleoside phosphonates (α-CNPs) are a class of structural mimics of natural 2′-deoxynucleotide 5′-triphosphates that were conceived to enable direct inhibition of viral DNA polymerases without the need for prior metabolic activation. This concept is best illustrated by cyclopentyl α-CNPs containing either a pyrimidine or purine base (**43a–e**, Figure 4), whose ability to be efficiently recognized and strongly inhibit HIV-1 reverse transcriptase (IC_50_ = 0.19–4.3 μM) was demonstrated in a cell-free HIV-1-RT assay (Keane et al., 2015; Balzarini et al., 2017). Both the phosphonate and carboxyl groups were found to be required for activity, which resided in the L-enantiomer of the compounds.

Structural studies aimed to disclose the molecular interactions between α-CNPs and the target enzyme revealed that these compounds bound in the HIV reverse transcriptase binding site in a nucleobase-specific manner competing with an incoming dNTP, but did not undergo incorporation into a growing viral DNA chain (Balzarini et al., 2015) No antiviral activity was observed in cell culture for any of these compounds; this is most likely due to an inefficient uptake by virus-infected cells as a consequence of the presence of a strong negative charge (Keane et al., 2015). It is interesting to note that the substitution of the cyclopentane ring with a flexible acyclic butenyl moiety shifted the selectivity spectrum of α-CNPs from HIV- toward herpesvirus-encoded DNA polymerases (John et al., 2015).

## Antitumoral Nucleoside Phosphonates

The clinical indication of nucleoside phosphonates is not restricted to the treatment of viral infections, but also include, albeit to a comparably much less exploited extent, anticancer chemotherapy. Preclinical studies in a number of murine models demonstrated the additional potential of ANP antiviral therapeutics (*S*)-HPMPC (**1a**) and PMEA (**18a**) to induce widespread apoptosis or to inhibit the growth of choriocarcinoma (Hatse et al., 1998), nasopharyngeal (Neyts et al., 1998), and cervical (Andrei et al., 1998b) carcinoma, as well as vascular tumors (Liekens et al., 1998, 2001). However, these compounds were not further pursued for clinical development against cancer.

On the other hand, the guanine congener of PMEA, i.e., PMEG (**44**, Figure 4), exhibited a remarkably potent *in vitro* cytostatic and antiproliferative activity against leukemic and solid tumor cell lines (Paborsky et al., 1997; Franek et al., 1999) including certain types of HPV-transformed cancer cells (Andrei et al., 1998a), which translated in a unique therapeutic profile. Its antitumor activity was confirmed *in vivo* using transplantable murine tumor models of lymphocytic leukemia (P388) and melanoma (B16) (Rose et al., 1990). In addition, PMEG effectively suppressed the growth of proliferative lesions induced by cotton tail papillomavirus (CRPV) and human papillomavirus type 11 (HPV-11) infections of rabbits and human foreskin xenografts in athymic mice, respectively (Kreider et al., 1990). However, the narrow safety margins observed upon exposure to **44** (Kreider et al., 1990) and high prevalence of kidney and gastrointestinal tract toxicities combined with the typically poor cellular permeability of ANPs posed serious limitations to its practical utility as anticancer agent. Therefore, PMEG was replaced in preclinical studies by its modified 2,6-diaminopurine prodrug *N*^6^-cyclopropyl PMEDAP (**46**, cPrPMEDAP, Figure 4), which proved equally cytostatic *in vitro* against a variety of tumor cell lines (Hatse et al., 1999), while allowing for higher antitumor efficacy and selectivity in a rat choriocarcinoma tumor model (Naesens et al., 1999). By virtue of its susceptibility to enzymatic deamination, cPr-PMEDAP can in fact undergo intracellular conversion to **44**, while minimizing toxicity due to diminished plasma exposure to PMEG. PMEG was shown to act via its diphosphate form PMEGpp formed upon cellular kinase phosphorylation, which was found to be a potent inhibitor of human polymerases α, δ, and ε involved in cellular DNA replication (Kramata et al., 1996). *In vitro*, PMEGpp was described to be efficiently recognized and incorporated into growing DNA chains by these polymerases (Cihlar and Chen, 1997; Pisarev et al., 1997; Kramata et al., 1998), resulting in termination of DNA synthesis with consequent arrest of cell division in S phase followed by induction of apoptosis. Resistance to both PMEG and cPr-PMEDAP was associated with a decreased capacity of the resistant cells to phosphorylate PMEG upon introduction of point mutations in the enzyme guanylate kinase (Mertlikova-Kaiserova et al., 2011).

The next step in prodrug design was carried out at Gilead Sciences with the synthesis of two bis-phosphonoamidate prodrugs, named GS-9191 (**47**) and GS-9219 (**48**, VDC-1101, Tanovea®, generic name: rabacfosadine), in an attempt to balance increased cellular penetration with tissue specific intracellular delivery and metabolic activation.

GS-9191 bearing a highly lipophilic bis(L-phenylalanine isobutylester)amidate moiety was purposefully prepared for topical skin administration with the aim of promoting selective accumulation of the active metabolite PMEGpp in the epithelial layer (Wolfgang et al., 2009). The mechanism of conversion of GS-9191 to PMEGpp was elucidated and found to be initiated in lysosomes by the carboxypeptidase cathepsin A (CatA)-mediated hydrolysis of a carboxylester bond of one of the amidate moieties (Birkus et al., 2011). The unmasked carboxyl group further displaces the L-phenylalanine isobutyl ester of the other amidate moiety, followed by release of cPrPMEDAP from cPrPMEDAP-Phe upon either enzymatic or pH dependent spontaneous cleavage. In subsequent steps, the cPr-PMEDAP metabolite undergoes translocation to the cytosol where further deamination most likely by *N*^6^-methyl-AMP aminohydrolase (Schinkmanova et al., 2006) and phosphorylation occur.

GS-9191 exerted a markedly more potent *in vitro* antiproliferative effect compared to its metabolic products cPrPMEDAP or PMEG against a broad spectrum of cervical carcinoma cell lines transformed by human papillomavirus (HPV) with an EC_50_ as low as 0.03 nM. Generally, non-HPV-infected and primary cells were less sensitive to the compound with EC_50_ values ranging between 1 and 15 nM (Wolfgang et al., 2009). In the cottontail rabbit papillomavirus (CRPV) infection model, topical administration of GS-9191 led to a dose-dependent decrease in the size of HPV-induced lesions, with cure achieved at the highest dose (0.1%) after 5 weeks. A topical ointment formulation of GS-9191 proved to be well-tolerated and clinically effective for the treatment of genital warts in a Phase II trial (ClinicalTrials.gov Identifier: NCT00499967, Recruitment status: completed). Graceway Pharmaceuticals, 6 November 2009 posting date. Graceway Pharmaceuticals acquires worldwide rights to GS 9191 from Gilead Sciences. Graceway Pharmaceuticals, Bristol, TN. http://www.gracewaypharma.com/news/0035-graceway-pharmaceuticals-acquires-worldwide-rights-gs-9191-gilead-~sciences).

The relatively more hydrophilic bis(L-alanine ethyl ester)amidate prodrug derivative GS-9219 was proposed to undergo a similar stepwise conversion pathway as GS-9191 leading to the intracellular release of PMEGpp. In this case, the adenosine deaminase like (ADAL) protein was shown to be primarily involved in the deamination step of cPr-PMEDAP to PMEG, since mutations affecting the enzyme activity promoted resistance to both GS-9219 and its cPr-PMEDAP metabolite (Frey et al., 2013).

GS-9219 displayed a preferential *in vitro* antiproliferative activity against activated lymphocytes and tumor cells of hematopoietic origin (EC_50_ = 27–1,043 nM) vs. quiescent lymphocytes (EC_50_ = 17.2 μM) and solid tumor cell lines (EC_50_ > 10 μM) (Reiser et al., 2008). A substantial *in vivo* efficacy combined with either no or low-grade adverse events was also shown in a series of preclinical and phase I/II studies in pet dogs with advanced-stage non-Hodgkin's lymphoma (NHL, 79% antitumor response rate) (Reiser et al., 2008; Vail et al., 2009), multiple myeloma (81% antitumor response rate) (Thamm et al., 2014), and cutaneous T-cell lymphoma (45% antitumor response rate) (Morges et al., 2014). Eventually, GS-9219 was licensed for clinical use in the systemic treatment of hematological malignancies in canines, and showed particularly useful in treating naïve multicentric (Thamm et al., 2017) and relapsing B-cell lymphoma (Saba et al., 2018). Based on these encouraging results, GS-9219 was also selected for evaluation in humans, but unfortunately failed to show an acceptable safety profile in a dose-escalating Phase I/II study involving patients with relapsed or refractory non-Hodgkin's lymphoma (NHL), chronic lymphocytic leukemia (CLL), and multiple myeloma (MM) (ClinicalTrials.gov Identifier: NCT00499239; Recruitment status: completed).

Subsequent work included the description of two additional prodrug analogs identified as GS-343074 and GS424044, whose structures have not been disclosed. In contrast to the activity spectrum of GS-9191, these compounds displayed considerable *in vitro* antiproliferative activity against multiple canine solid cancer cell lines, and seemed to favor a mechanism of action based on a cytotoxic rather than cytostatic effect upon induction of cancer cell apoptosis (Lawrence et al., 2015).

Finally, it should be mentioned that the alkoxyalkyl prodrug approach developed by Hostetler was also applied to PMEG, and ODE-PMEG **45** especially turned out to exert significant antiproliferative activity *in vitro* against different human cervical carcinoma cell lines (Valiaeva et al., 2010). In a murine model, intratumoral injection of 25 μg of **45** daily for 21 days resulted in near complete disappearance of measurable tumors [vs. 100 μg of ODE-(S)-HPMPC **5b**], suggesting that ODE-PMEG may find use for local or topical treatment of cervical dysplasia.

## Antibacterial Nucleoside Phosphonates

**Figure 5:**
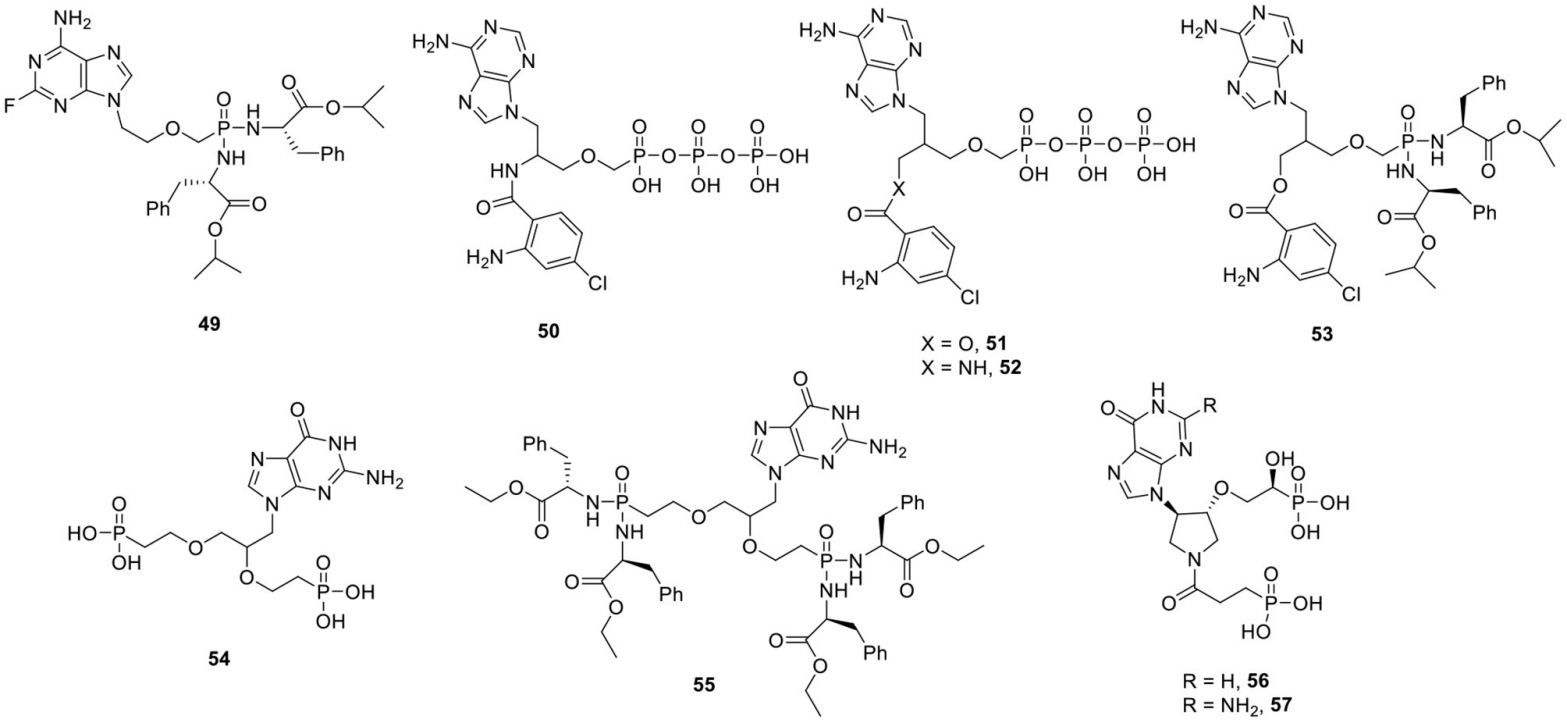
Antibacterial adenylyl cyclase inhibitors and HGPRT inhibitors.

The edema factor (EF) is an important virulence factor from *Bacillus anthracis*. Adefovir diphosphate is a potent inhibitor of the adenylyl cyclase activity of EF with high affinity (Ki = 27 nM). Another bacterium, *Bordetella pertussis*, secretes several virulence factors, and among them, CyaA is an adenylyl cyclase toxin (ACT) that is a tight binder of adefovir diphosphate. Based on the adefovir-diphosphate ACT crystal structure, a number of 2-substituted PMEA analogs were prepared and converted to their isopropyl ester bis(L-phenylalanine)bisamidate prodrugs. All the bisamidate prodrugs exhibited overall lower activity than the positive control adefovir dipivoxil when tested for their ability to inhibit ACT activity in macrophages. The 2-fluoro derivative **49** (Figure 5) emerged as the most potent in this series with an IC_50_ of 0.14 μM for the bacterial AC, whereas it has no effect on mammalian ACs. In addition, this compound lacked cytotoxicity against a panel of cancer cell lines and also did not display any toxicity toward normal cells (Cesnek et al., 2015).

A small series of racemic acyclic nucleoside diphosphophosphonates bearing a 5-chloro-anthraniloyl substituent attached through various linkers to the acyclic chain were prepared (Figure 5) and evaluated as potential inhibitors of the AC activity of the EF from *Bacillus anthracis*. In this case, compound **50** showed the best inhibitory activity (IC_50_ = 69 nM), followed by **51** (IC_50_ = 613 nM) and **52** (IC_50_ = 140 nM). As these three diphospho-phosphonate analogs are potent inhibitors of AC in a cell-free, biochemical assay, the corresponding lipophilic isopropyl ester bis(L-phenylalanine) prodrugs were synthesized. Only the prodrug **53**, derived from the parent compound **51** showed modest inhibition of AC activity in murine macrophages, with an IC_50_ value of 12 μM, whereas other prodrugs lacked activity.

Hypoxanthine-guanine phosphoribosyltransferase (HGPRT) catalyzes the formation of the 6-oxopurine nucleoside monophosphates, IMP and GMP from 5-phospho-α-D-ribosyl-1-pyrophosphate and hypoxanthine or guanine. It is an important enzyme in the purine salvage pathway and it has been shown to be essential for the growth of *Mycobacterium tuberculosis*, the causative agent of tuberculosis. A series of various acyclic nucleoside phosphonates with a 6-oxo-purine nucleobase (either guanine or hypoxanthine) was synthesized. The most potent inhibitor was the bisphosphonate analog **54**, displaying a Ki value of 0.69 μM (Figure 5). Unfortunately, this compound lacks selectivity, as it is equally active against the human HGPRT. For evaluation in cellular assays, the lipophilic ethyl L-phenylalanine phosphonoamidate prodrug **55** was prepared, that showed anti-tuberculosis activity against a virulent strain of *M. tuberculosis* (H37Rv), with an IC_50_ value of 9 μM (Eng et al., 2015).

Pyrrolidine bisphosphonates (Figure 5) with either a hypoxanthine or guanine nucleobase (compounds **56** and **57**, respectively) were found to be very potent inhibitors of mycobacterial HGPRT (Ki values for both analogs was 60 nM). Unfortunately, both derivatives are 20- to 60-fold more potent against human HGPRT with a Ki value of 1 and 3 nM, respectively (Eng et al., 2018).

**Figure 6:**
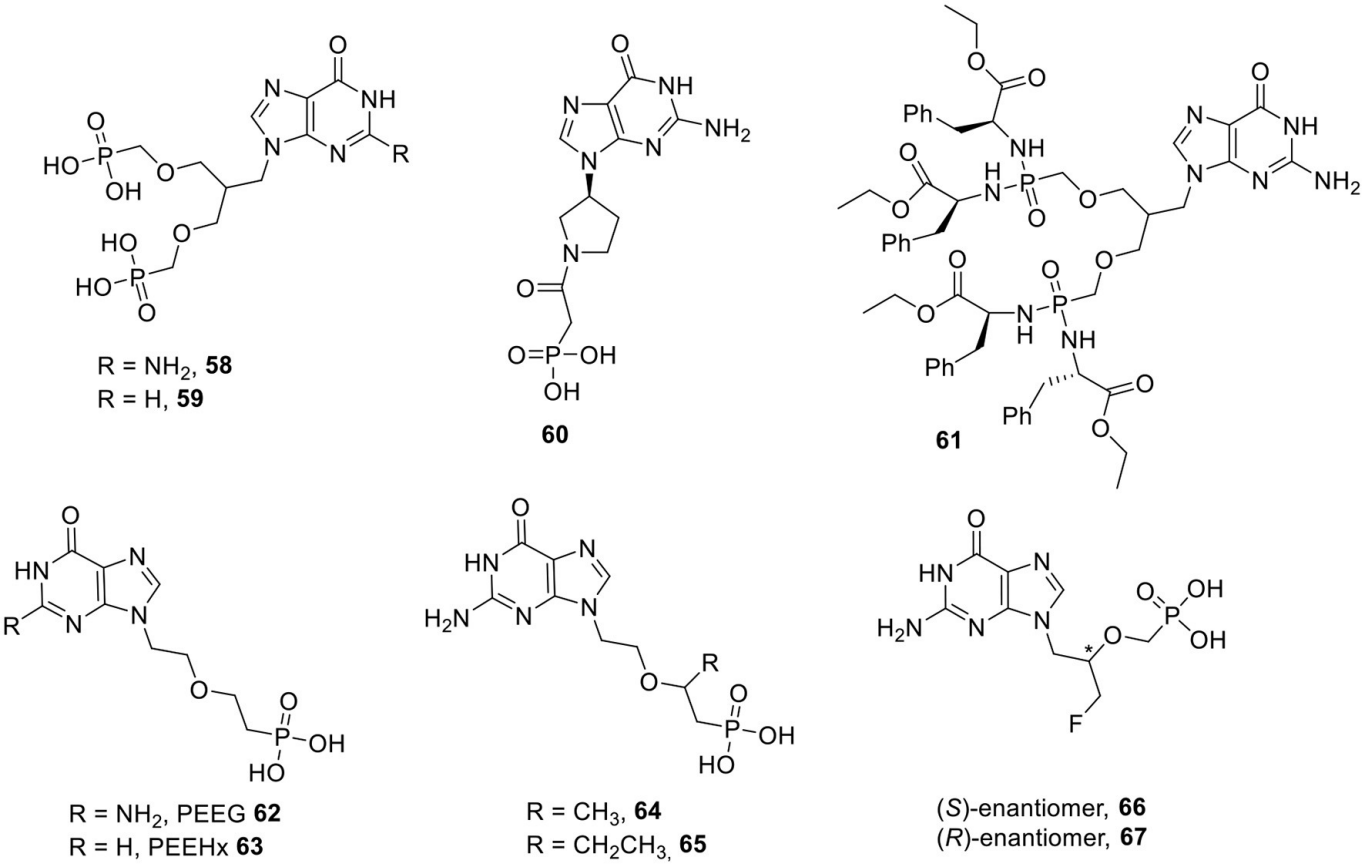
I Inhibitors of bacterial PRTases and inhibitors of HGXPRT of *P. falciparum*.

Bacteria, such as *E. coli*, possess two 6-oxopurine phosphoribosyltransferases (PRTases): a xanthine-guanine phosphoribosyltransferases (XGPRT) and a hypoxanthine PRTase (HPRT). The introduction of a second phosphonate group on a 2-(phosphonoethoxy)ethyl (PEE) side chain yielded compound **58** (Figure 6) with very high affinity for EcXGPRT (Ki = 10 nM). Replacing the guanine nucleobase with hypoxanthine affords the nucleoside phosphonate analog **59** which is a good inhibitor of HPRT (Ki = 0.8 μM). To confer rigidity to the acyclic, flexible linker moiety and possibly tighter binding, cyclic pyrrolidine derivatives of guanine were prepared, from which compound **60** emerged as the most potent congener. In order to mask the negative charges of the phosphonate groups and thus to increase cell permeability, prodrugs were synthesized. It was shown that the phosphonoamidate prodrug **61** arrested the growth of *M. tuberculosis* in cell culture, with IC_50_ values of 5 μM, and lacked cytotoxicity against a human lung carcinoma cell line (A549) (Keough et al., 2013a).

## Antiparasitic Nucleoside Phosphonates

There are four *Plasmodium* species responsible for human malaria: *falciparum, vivax, ovale*, and *malariae*. *P. falciparum* (Pf) is the cause of the most lethal form of malaria. Hypoxanthine-guanine-xanthine phosphoribosyltransferase (HGXPRT) catalyzes the formation of the 6-oxopurine nucleoside monophosphates, inosine monophosphate (IMP), guanosine monophosphate (GMP) and xanthosine monophosphate (XMP), from 5-phospho-α-D-ribosyl-1-pyrophosphate and hypoxanthine, guanine, or xanthine. It is an important enzyme in the purine salvage pathway because parasites of the genus *Plasmodium* lack the *de novo* pathway for purine nucleotide biosynthesis.

The first, structurally simple, acyclic nucleoside phosphonates that were identified as inhibitors of PfHGXPRT were 9-[2-(2-phosphonoethoxy)ethyl]guanine **62** (PEEG) and 9-[2-(2-phosphonoethoxy)ethyl]hypoxanthine **63** (PEEHx), that displayed Ki values against PfHGXPRT of 0.1 and 0.3 μM, respectively (Figure 6). In addition, both compounds are selective for the malaria enzyme as the Ki values for the human HGPRT are 10-fold higher (Keough et al., 2009).

The effect of branching of the phosphonoethoxyethyl chain on the inhibition of PfHGXPRT and human HGPRT was studied by adding small alkyl chains either to the carbon attached directly to the phosphorus atom (α-branched derivatives) or to the adjacent carbon (β-branched derivatives). The α-branched derivatives completely lacked HG(X)PRT (human as well as malaria), whereas among the β-substituted derivatives, especially the guanosine congeners are active. The methyl and ethyl substituted analogs (compounds **64** and **65**) are endowed with Ki values for PfHGXPRT of 1 and 5 μM, respectively. Remarkably, these β-branched derivatives bind 10 times more strongly to the human enzyme, when compared to the *P. falciparum* counterpart (Hocková et al., 2009).

**Figure 7:**
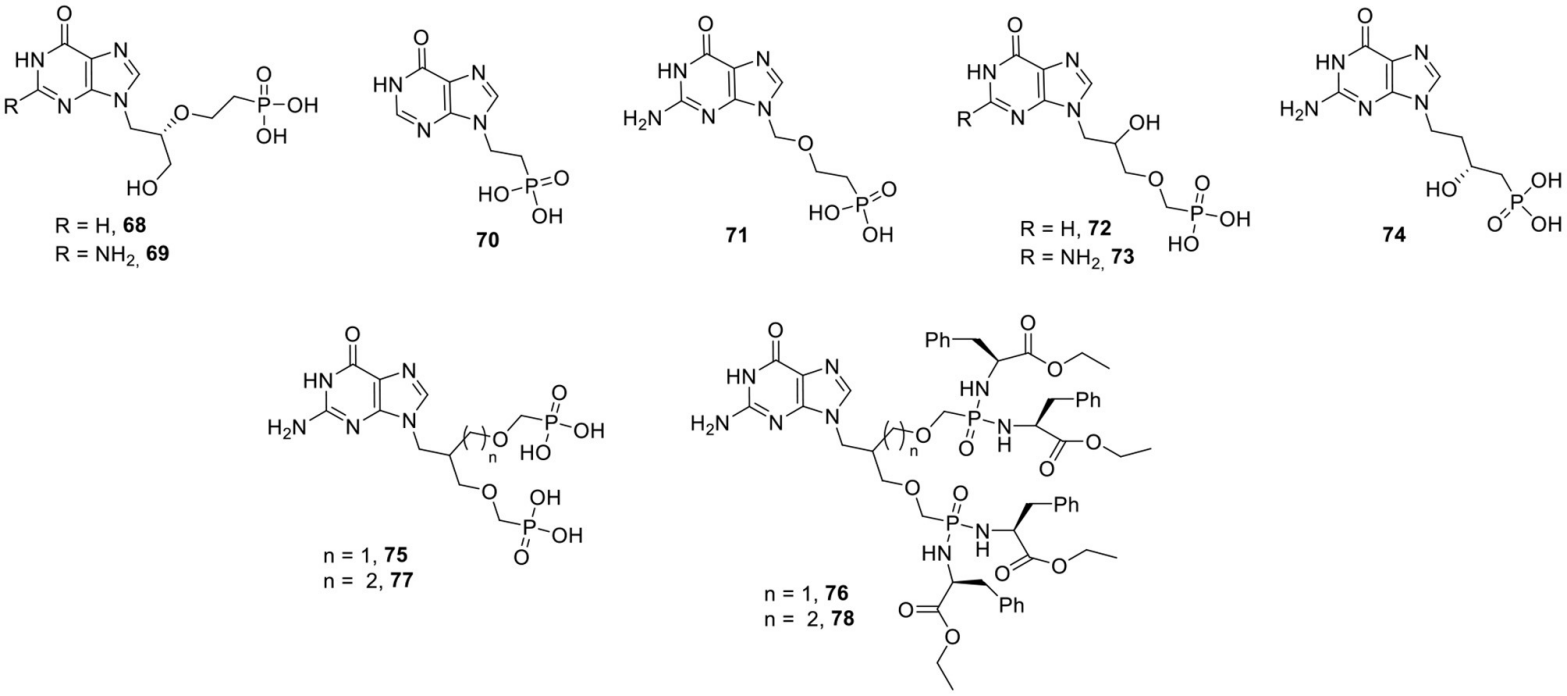
ANPs as inhibitors of HGXPRT of *P. falciparum*.

The addition of a fluoromethylene group on the PEE linker created 3-fluoro-(2-phosphonoethoxy)propyl derivatives having a chiral center (Figure 6). Both enantiomers were equally active against PfHGXPRT (Ki = 1 μM), although their affinity for the PvHGXPRT and the human HGPRT was different. The (*S*)-enantiomer **66** showed modest activity for PvHGXPRT (Ki = 3.4 μM) and was quite potent as human HGXPRT inhibitor (Ki = 0.1 μM). The (*R*)-enantiomer **67** lacked activity for PvHGXPRT (Ki = 29 μM) and was moderately active as human HGPRT inhibitor (Ki = 4.7 μM) (Baszczynski et al., 2013).

(*S*)-3-Hydroxy-2-(phosphonoethoxy)propyl (HPEP) derivatives with a hypoxanthine and guanine nucleobase (compounds **68** and **69**, respectively) were synthesized (Figure 7). Both analogs did inhibit PfHGXPRT (Ki = 2 μM for the hypoxanthine analog **68** and 0.1 μM for the guanine derivative **69**), but also affected the human HGPRT (Ki = 0.5 and 0.6 μM, for the hypoxanthine and guanine analog, respectively) (Kaiser et al., 2015). Further SAR studies focused on structural variation of the acyclic linker. Shortening the PEE linker usually gave a decreased activity, although one particular analog, with a two carbon atom linker yielded compound **70**, which is only slightly less active as PfHGXPRT inhibitor (Ki = 2.2 μM), but has a diminished selectivity profile (Ki value for the human enzyme = 3.4 μM). Changing the position of the oxygen in the acyclic part of PEEG afforded compound **71** having a Ki of 5 μM vs. PfHGXPRT (Cesnek et al., 2012). A number of derivatives with a 2-hydroxy-3-(phosphonomethoxy)propyl side chain also have been prepared. The hypoxanthine congener **72** inhibited both PfHGXPRT and PvHGPRT with Ki values of 2 and 5 μM, respectively, but did not inhibit the human enzymes at a concentration of 30 μM. In contrast, the guanosine analog **73** had a Ki value of 1.4 and 10 μM for PfHGXPRT and PvHGPRT, respectively, and was equally active against the human enzyme (Ki = 4 μM) (Krečmerová et al., 2012).

Very recently, a series of β-hydroxy-ANPs with different chain lengths was prepared (Figure 7). Among a set of 24 derivatives, a phosphonate with a guanine nucleobase, a *n*-butyl linker, and a β-hydroxy group with the *R*-configuration, was the most promising one (compound **74**). Remarkably, the free phosphonate displayed potent activity in a cellular assay when tested against *P. falciparum* infected red blood cells (IC_50_ = 74 nM). This compound was equally active against multidrug resistant and chloroquine resistant *P. falciparum* strains. In addition, it is completely devoid of cytotoxicity when tested against a human cancer cell line (K562) (Cheviet et al., 2020).

Guided by available X-ray crystal structures, a new series of ANPs, containing a second phosphonate group, designed to occupy an additional pocket in the enzyme, were prepared (Figure 7). Compound **75** turned out to be a very potent inhibitor of PfHGXPRT (Ki = 0.07 μM) and PvHGPRT (Ki = 0.6 μM). This compound was also endowed with potent inhibition of human HGPRT with a Ki value of 0.03 μM. These bisphosphonates were highly polar and unable to cross the cell membranes. Therefore, a phosphoramidate-type prodrug was synthesized to increase its antimalarial activity. This prodrug **76** did show activity against different *P. falciparum* strains with IC_50_ values in the 10 μM range (Keough et al., 2013b). The insertion of an additional methylene linker afforded compound **77**, which was a very potent inhibitor of the human enzyme (Ki = 0.006 μM), with still very good affinity for PfHGXPRT (0.07 μM). The corresponding Protide **78** was able to inhibit the growth of *P. falciparum* (IC_50_ between 5 and 7 μM) and was devoid of cytotoxicity.

**Figure 8:**
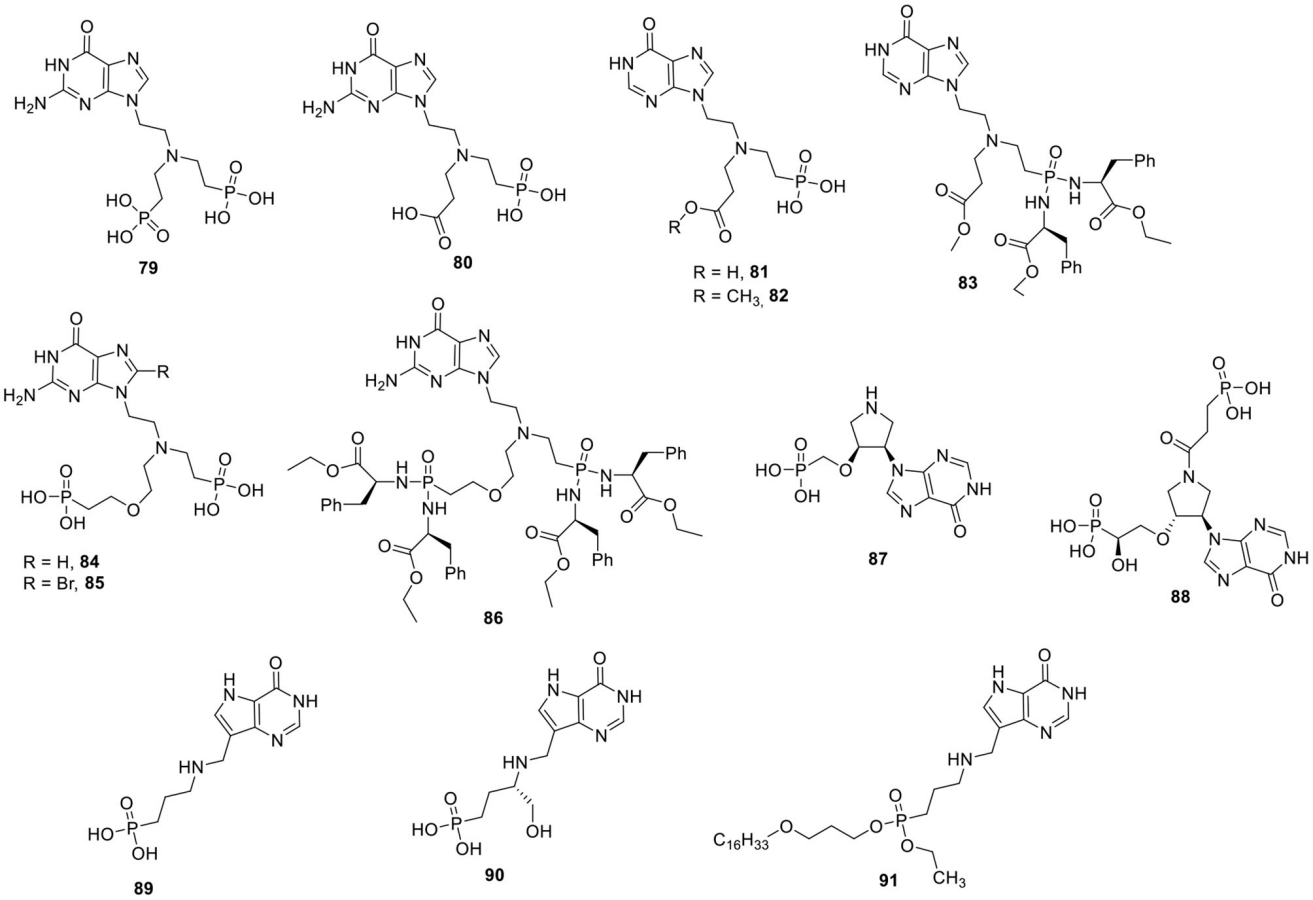
Aza-ANPs as inhibitors of HGXPRT.

In an effort to increase affinity and/or selectivity for PfHGXPRT and PvHGPRT vs. human HGPRT, the oxygen in the phosphonate side chain was replaced by a nitrogen atom, yielding an aza-ANP (Figure 8). When two phosphonate groups were connected via an ethyl linker to this central nitrogen, and having a guanosine nucleobase, a potent, but non-selective inhibitor was obtained (compound **79**), with Ki values for human HGPRT, PfHGXPRT, and PvHGPRT of 0.2, 0.3, and 0.9 μM, respectively.

Replacing one of the phosphonate group by a carboxylic acid as an alternative ionizable moiety yielded compound **80** which was especially active against PvHGPRT, having a Ki value of 50 nM. A number of hypoxanthine based analogs, either having a terminal carboxylic acid (compound **81**) or carboxylic acid methyl ester group (compound **82**), were discovered that show promising selectivity for PfHGXPRT (Ki values of 0.4 and 0.1 μM, respectively), lacking activity for the human counterpart (Ki > 200 μM) (Hockova et al., 2012). A prodrug of **82** (compound **83**) was able to arrest the growth of *P. falciparum* in cell culture and did not display cytotoxicity when evaluated against different human cancer cell lines (Hockova et al., 2015).

In a follow-up study, a new series of aza-ANPs with a central nitrogen atom substituted with two different phosphonate containing side chains, was prepared (Figure 8). The guanine based analog **84** was a potent compound with Ki values of 0.19 and 0.03 μM against PfHGXPRT and PvHGPRT, respectively. When 8-bromoguanine was installed as nucleobase, a more potent compound **85** was obtained with a Ki value of 0.08 μM (PfHGXPRT) and 0.01 μM (PvHGPRT). Unfortunately, both derivatives also displayed potent inhibition of the human HGXPRT with Ki values of 0.08 and 0.04 μM, for the guanine and 8-bromo-guanine congener, respectively. A phosphoramidate prodrug **86** was able to suppress *P. falciparum* replication in cultured erythrocytes (IC_50_ = 6 μM), and lacked cytotoxicity against human cells (Keough et al., 2015).

The conformational lock of the nitrogen-containing side chain was achieved by the synthesis of a series of pyrrolidine phosphonate analogs (Figure 8). The limited SAR demonstrated that the stereochemistry of the carbon atom bearing the phosphonomethoxy group and the nature of the nucleobase determines HGPRT inhibition. Compound **87** was the most potent with a Ki value of 0.6 μM (*P. falciparum*). Importantly, this compound did not have any affinity for the human enzyme (Pohl et al., 2015). A novel series of pyrrolidine nucleoside bisphosphonates (PNBPs) was recently published (exemplified by compound **88**) with low nM activity for the various HGPRT enzymes (Ki values of 6, 2, and 1 nM for the PvHGPRT, PfHGXPRT, and human HGPRT, respectively) (Keough et al., 2018).

Structurally similar to the azaANPS are the acyclic immucillin phosphonates (AIPs). A typical structure feature of this compound family is the presence of 9-deaza-hypoxanthine as nucleobase and hence these compounds can be considered as acyclic *C*-nucleoside phosphonates. Compounds **89** and **90** are both competitive inhibitors of PfHGXPRT with Ki values of 10.6 and 0.65 nM, respectively. Both analogs displayed much lower affinities for the human enzyme with Ki values of 4,940 and 385 nM. Hence, these AIPs are more than 400-fold selective for the parasite enzyme. As these free phosphonates did not show activity against cultured parasites, a series of lysophospholipid prodrugs were synthesized from which analog **91** was the most potent one, as it inhibited the growth of various *P. falciparum* strains with an IC_50_ in the 2 μM range (Hazleton et al., 2012; Clinch et al., 2013).

**Figure 9:**
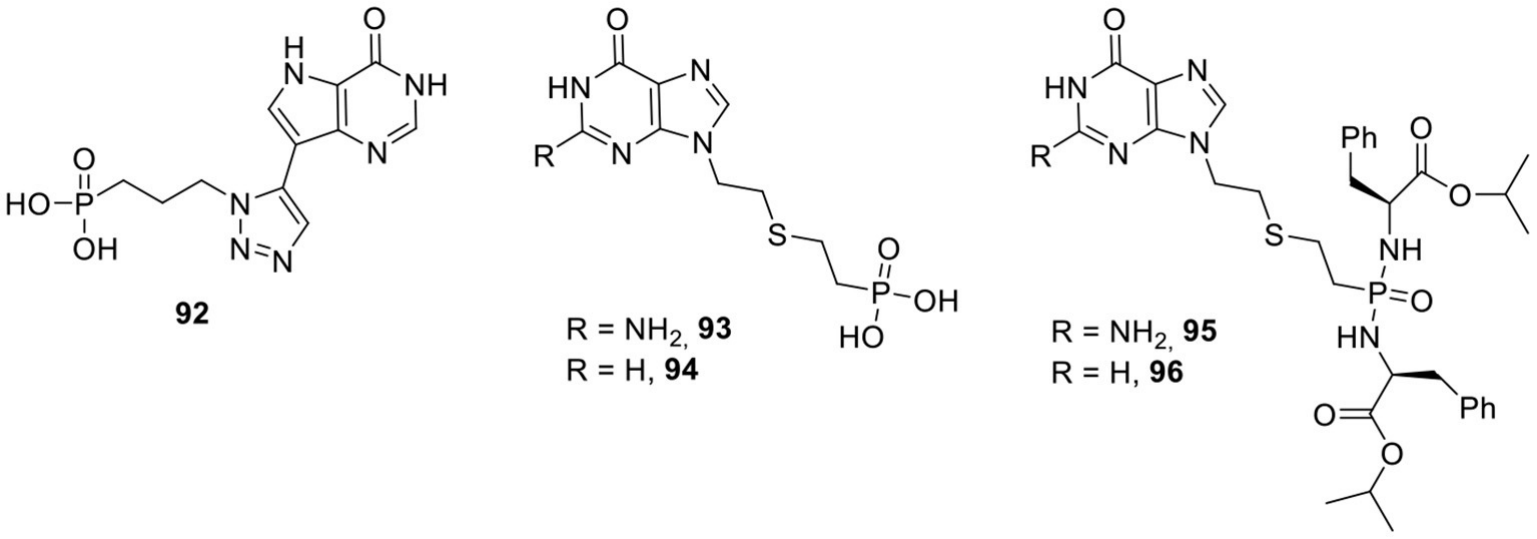
Triazole-based and Thia-ANPs.

The introduction of rigidity into the acyclic chain of AIPs was achieved by the insertion of heterocyclic structures between the 9-deazahypoxanthine group and the phosphonate moiety (Figure 9). Among a series of 18 compounds, triazole derivative **92** was the most potent with Ki values of 2 and 1 μM for PfHGXPRT and PvHGPRT, respectively, and no inhibition of the human enzyme was observed at 71 μM (Kaiser et al., 2017).

Recently, thia-ANPs were described containing a sulfur in various oxidation states (sulfide, sulfoxide, and sulfone) and at different positions of the acyclic chain (Figure 9). The most potent congeners from this series (compounds **93** and **94**) are the direct counterparts from PEEG and PEEHx, respectively, with similar binding data to PfHGXPRT. The corresponding phosphoramidate prodrugs **95** and **96** do show antimalaria effects in cell culture with IC_50_ values in the 4–6 μM range.

## Nucleoside Phosphonates Interfering With Purinergic Signaling

Extracellular nucleotides function as signaling molecules through the activation of nucleotide receptors. These receptors are referred to as purinergic P2 receptors. In contrast to P1 receptors, which are activated by the nucleoside adenosine, P2 receptors are activated by nucleotides. On the basis of their signaling properties, P2 receptors can be further subdivided into metabotropic P2Y receptors that belong to the rhodopsin-like branch of family A G-protein coupled receptors, and ionotropic P2X receptors that are nucleotide-gated ion channels.

Two subfamilies are distinguished within the P2Y receptors. P2Y1, P2Y2, P2Y4, P2Y6, and P2Y11 receptors are referred to as the P2Y1-like subfamily, whereas the P2Y12, P2Y13, and P2Y14Rs belong to the P2Y12-like subfamily. The P2Y receptor family is activated by either or both adenine and uracil nucleotides. Adenosine-5′-diphosphate (ADP) is the preferred natural agonist of P2Y1, P2Y12, and P2Y13 receptors, whereas uridine-5′-diphosphate (UDP) is the physiological agonist of P2Y6 and P2Y14. Other P2 receptors are preferentially activated by nucleoside-5′-triphosphates. Adenosine-5′-triphosphate (ATP) binds and activates P2Y2, P2Y4, and P2Y11, whereas uridine-5′-triphosphate (UTP) functions as the natural agonist of P2Y2 and P2Y4 receptors. P2X receptors are further subdivided into P2X1-7 receptors with the natural, endogenous agonist of these P2X receptors being ATP.

The natural ligands of the P2 receptors are highly charged nucleoside phosphates. Although these nucleotides are usually hydrolytically stable in the pH range of about 4–11, they are metabolically labile, as they are rapidly degraded in biological environment by enzymatic hydrolysis. Therefore, besides structural modification of the natural nucleoside-di-, and triphosphates, medicinal chemistry programs focused on the synthesis of nucleoside phosphonates as metabolically stable analogs of the natural agonists.

### P2X Receptor Ligands

**Figure 10:**
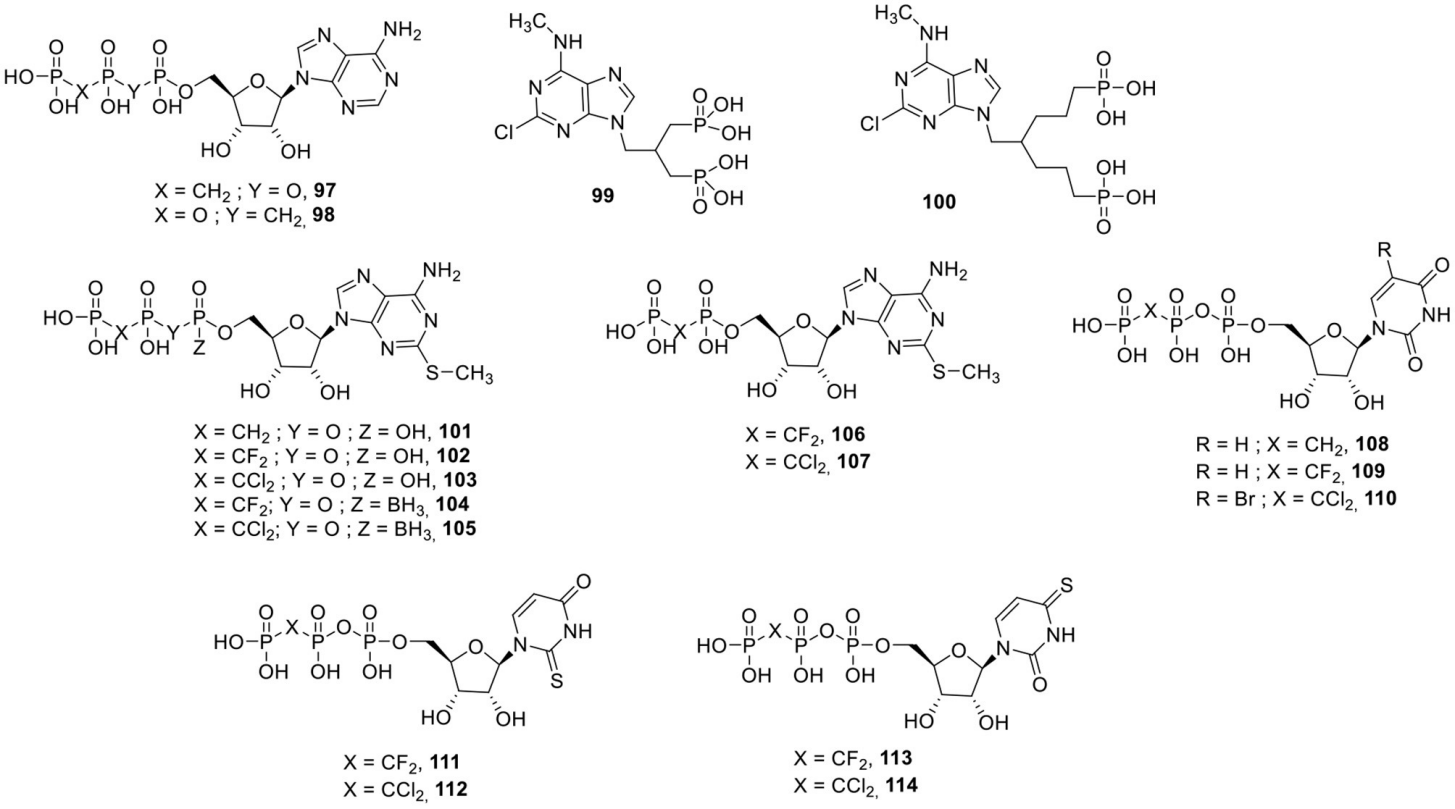
P2X, P2Y1, and P2Y2 receptor ligands.

P2X receptors are ion channels that are permeable for various cations (sodium, potassium and calcium). Since these receptors are activated by ATP, most P2X receptor agonists are based on structural variation of ATP. Analogs in which methylene groups replaced oxygen atoms of the phosphate chain of ATP are known to act as P2X agonists (Figure 10). α,β-Methylene-adenosine-triphosphate (compound **97**) acts preferentially on P2X1 and P2X3 receptors, with somewhat lower potency at the P2X4 receptor. β,γ-methylene-ATP (compound **98**) was the most potent at the P2X1 receptor, lacking activity for other P2X receptors. Although agonists, as well as antagonists, based on different types of chemistry are known for various other P2X receptors, none of them is based on a nucleoside phosphonate scaffold.

### P2Y Receptor Ligands

#### P2Y1 Receptor Ligands

A few acyclic nucleoside phosphonates were found to act as P2Y1 antagonists (Figure 10). MRS2496 (compound **99**) acted as an inhibitor of ADP-induced activation of rat platelets with a Ki value of 0.68 μM. The longer chain homolog **100** was much less active (Ki = 25 μM), indicating a crucial role of the spacer length (Xu et al., 2002). Later on, it was demonstrated that MRS2496 had strong binding affinity for the P2Y1 receptor as measured by a radioligand-based binding assay, with a Ki value of 0.76 μM and that it also inhibited the ADP-induced aggregation of human platelets with an IC_50_ values of 62.8 nM (Cattaneo et al., 2004).

Several 2-thiomethyl ATP analogs were synthesized as P2Y1 agonists (Figure 10) (Eliahu et al., 2009). The most potent analog within this series carried a β,γ-methylene bridge (compound **101**) and had an EC_50_ value of 80 nM. The corresponding difluoromethylene derivative (compound **102**) was 10-fold less active as P2Y1 receptor agonist (EC_50_ = 0.76 μM), whereas the dichloromethylene analog (compound **103**) was 100-fold less active (EC_50_ = 9.54 μM). Replacing the non-bridging oxygen on the phosphorus with BH_3_ created an additional chiral center yielding compound and the diastereomers were separated. In case of the difluoromethylene analogs (compound **104**), one isomer lacked activity, whereas the corresponding diastereomer was modestly active as P2Y1 receptor agonist (EC_50_ = 2.13 μM). For the 2-thiomethyl-α-borano-β,γ-dichloromethylene ATP analog (compound **105**), one diastereomer displayed quite potent P2Y1R agonism (EC_50_ = 0.57 μM), with the other diastereomer being 2-fold less active (EC_50_ = 1.2 μM). As 2-thiomethyl ADP was a selective and highly potent P2Y1 receptor agonist, suffering from low chemical and enzymatic stability, the α,β-bridging oxygen was replaced with chemical and metabolically stable dihalomethylene moieties. The difluoro analog (compound **106**) was more potent than the dichloro counterpart (compound **107**) with EC_50_ values of 0.98 and 3.10 μM, respectively (Eliahu et al., 2010).

#### P2Y2 Receptor Ligands

Quite a number of P2Y2 agonists are known in literature, and most of them are derived from the natural agonists ATP and UTP. Most medicinal chemistry campaigns focused on UTP as lead structures due to the expected higher selectivity of pyrimidine nucleotides vs. the other P2 receptor subtypes that are activated by the adenine nucleotides.

β,γ-methylene UTP **108** showed weak activity as P2Y2 agonist (EC_50_ = 73 μM), although the corresponding β,γ-difluoromethylene analog **109** displayed an improved activity as P2Y2 agonist (EC_50_ = 4.9 μM), and especially the dichloromethylene congener showed promising P2Y2 agonism (EC_50_ = 0.612 μM). This dihalo modification was combined with 5-bromo-uracil as nucleobase, yielding the β,γ-dichloromethylene-5-bromo-UTP analog **110**, that was endowed with an EC_50_ value of 0.354 μM (Figure 10) (El-Tayeb et al., 2006).

As it was well-known that substitution of the 2-oxo group by a 2-thio functionality enhanced the affinity of UTP for the P2Y2 receptor, this structural modification has been combined with the introduction of phosphonate groups. 2-Thio-β,γ-difluoromethylene-UTP (compound **111**) and 2-thio-β,γ-dichloromethylene-UTP (compound **112**) are both endowed with low μM agonistic activity when tested against the P2Y2 receptor (EC_50_ values of 1.63 and 2.51 μM, respectively) (Ko et al., 2008). As an expansion of the SAR study, the dichloromethylene and difluoromethylene modification was combined with a 4-thio-uridine nucleobase, affording compounds **113** and **114**, that exhibited EC_50_ values of 1.81 and 0.134 μM, respectively (El-Tayeb et al., 2011).

**Figure 11:**
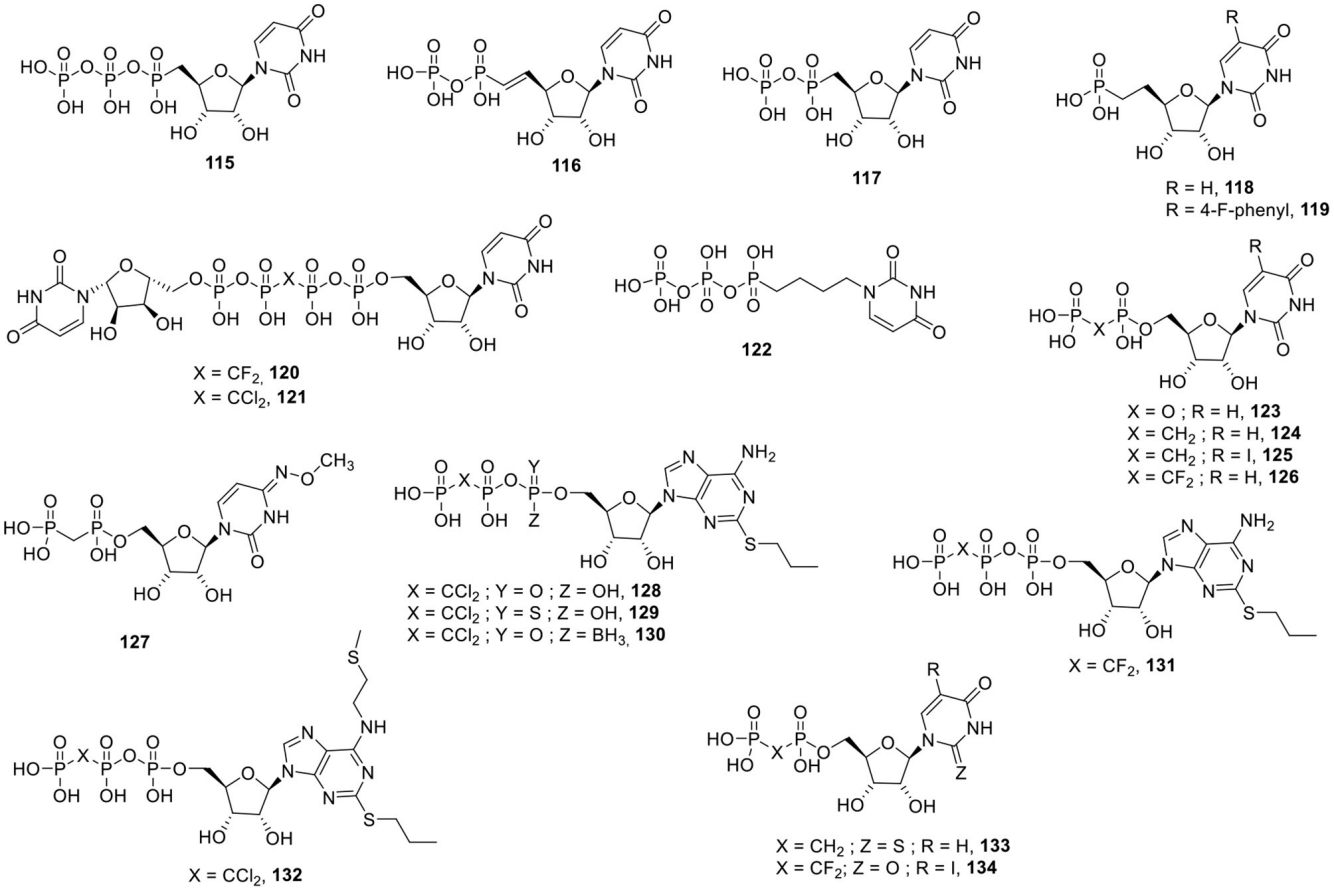
P2Y2, P2Y6, P2Y11, P2Y12, and P2Y14 receptor ligands.

Uridine-5′-(diphospho)-phosphonate **115** and uridine 5′-(diphospho)-vinylphosphonate **116** behaved as full agonists for the P2Y2 receptor, although the potencies were reduced by a factor 16–76, when compared to the natural ligand UTP (Figure 11). On the other hand, uridine 5′-phospho-phosphonate **117** and uridine 5′-methylene-phosphonate **118** both acted as partial agonists, with EC_50_ values of 3.4 and 1.6 μM, respectively (Cosyn et al., 2009). The 5′-methylene derivative **118** was selected as lead structure for further SAR exploration. It led to the discovery of a 5-(4-fluorophenyl) modified uridine 5′-phosphonate derivative **119**, that was a P2Y2 receptor agonist (EC_50_ = 0.4 μM), although the maximal effect observed was only 43% of that observed with UTP. Additional pharmacological profiling suggested that this compound activated the P2Y2 receptor in a allosteric manner (van Poecke et al., 2012).

Phosphonate analogs were also prepared as analogs of dinucleoside tetraphosphate derivatives (Figure 11). The insertion of a β,γ-difluoro- or β,γ-dichloro-methylene linker afforded compounds that were less active at the P2Y2 receptor when compared with the tetraphosphate analog (EC_50_ = 0.21 μM). The difluoro analog **120** (EC_50_ = 2.27 μM) was more potent than the dichloro analog **121** (EC_50_ = 7.77 μM), suggesting that it is a better mimic of the phosphate ester bond (Ko et al., 2008).

A series of analogs in which the ribose moiety of the natural uracil nucleotides was replaced by various acyclic ribose-mimetic structures was also prepared (Figure 11). These acyclic nucleotide analogs were devoid of any agonistic activity at the P2Y2 receptor, but, in contrast, behaved as weak antagonist with an IC_50_ value of 92 μM for compound **122** (Sauer et al., 2009).

#### P2Y6 Receptor Ligands

UTP analogs in which the phosphorus-β,γ-oxygen bridge was replaced by a dihalomethylene moiety were evaluated for P2Y6 agonism. β,γ-dichloromethylene-UTP showed an EC_50_ value of 6.2 μM at the P2Y6 receptor, but was more potent at the P2Y2 receptors (EC_50_ = 0.612 μM). On the other hand, this compound showed very high selectivity vs. the P2Y4 receptor (EC50 > 100 μM). The closely related β,γ-difluoromethylene-UTP analog (compound **109**) is 2-fold more potent at the P2Y6 receptor (EC_50_ = 2.63 μM), than at the P2Y2 receptor (EC_50_ = 4.9 μM), whereas it completely lacked agonistic activity at the P2Y4 receptor (EC_50_ > 100 μM), similarly as the dichloromethylene analog.

Based on these data, these phosphonate modifications were combined with structural variations of the uracil nucleobase. 5-Bromo-β,γ-dichloromethylene-UTP **110** was discovered to be a full agonist at the P2Y6 receptor, with an EC_50_ value of 120 nM (El-Tayeb et al., 2006). This UTP analog shows some selectivity for the P2Y6 receptor, as it is 33- and 3-fold less active against the P2Y4 and P2Y2 receptors, respectively. 2-Thio-β,γ-dichloromethylene-UTP **112** showed moderate potency at the P2Y6 (EC_50_ = 7.7 μM) and at the P2Y4 receptor (EC_50_ = 3.85 μM), but is especially attractive as a P2Y2 agonist (EC_50_ = 0.794 μM) (El-Tayeb et al., 2011).

The introduction of an α,β-methylene substituent into the natural P2Y6 agonist UDP **123** (EC_50_ = 0.30 μM) yielded analog **124** that was embarked with only a slight decrease in P2Y6 activity (EC_50_ = 0.66 μM). The corresponding 5-iodo congener **125** has a similar agonistic activity on the P2Y6 receptor (EC_50_ = 0.13 μM). Strikingly, the α,β-difluoromethylene analog **126** was completely inactive (Ko et al., 2008; Maruoka et al., 2010). The potency-enhancing 4-methoxyimino modification was combined with an α,β-methylene substitution yielding analog **127** which promising activity as a selective P2Y6R agonist (EC_50_ = 0.678 μM), lacking activity for the P2Y4 and P2Y2 receptors (EC_50_ > 10 μM).

#### P2Y11 Receptor Ligands

A number of nucleoside triphosphate mimics containing a dichloromethylene linker between the β- and γ-phosphate group and an *n*-propyl-thio substituent at position 2 of the adenine nucleobase were prepared (Figure 11). AR-C67085 (compound **128**) lacked antagonistic activity against the P2Y11 receptor, but was a potent agonist of the P2Y11 receptor. The EC_50_ values of AR-C67085 for IP3 and cyclic AMP accumulation were respectively 8.9 and 1.5 μM, as compared to respectively 72 and 17.4 μM for the natural agonist ATP (Communi et al., 1999).

The presence of a thio-group at the α-phosphate position yielded a chiral center (compound **129**). The S*p* diastereomer displayed an EC_50_ of 0.6 μM, whereas an EC_50_ of 0.85 μM was found for the R*p* isomer.

Substitution of the non-bridging oxygen atom of the α-phosphate group by a borano group yielded compound **130**. The EC_50_ values of 0.03 μM for the S*p* isomer and 0.1 μM for the R*p* diastereomer indicated a diastereoselective preference of the P2Y11 receptor (Haas et al., 2013).

#### P2Y12 Receptor Ligands

Among the P2 receptors, P2Y12 has been studied most extensively, because of its proven value as drug target for the development of antithrombotic drugs. AR-C66096 (compound **131**) and AR-C67085 (compound **128**) are both ATP mimics with a dihalomethylene bridged 5′-triphosphate moiety and a thiopropyl group at position 2 of the adenine ring. Both analogs inhibit the ADP induced aggregation of blood platelets, which is consistent with P2Y12 antagonism. A structurally related analog (compound **132**, AR-C69931, Cangrelor) received marketing approval as an intravenous P2Y12 inhibitor for the treatment of thrombosis.

#### P2Y14 Receptor Ligands

Replacement of the bridging oxygen of the diphosphate group of UDP (compound **123**) with a methylene (compound **124**) or difluoromethylene group (compound **126**) was well-tolerated for agonistic activity at the P2Y14 receptor, resulting in high potency compounds endowed with EC_50_ values of 11 and 63 nM, respectively. Both compounds also show good activity at the P2Y6 receptor. Combining a carbon bridge with a known potency-enhancing uracil modification resulted in in 2-thio-α,β-methylene analog (compound **133**) that displayed excellent potency at the P2Y14 receptor with an EC_50_ value of 0.92 nM (Figure 11). Combining the α,β-difluoromethylene and 5-iodo-uracil moiety afforded compound **134** with good agonistic activity at the P2Y14 receptor (EC_50_ = 142 nM) (Das et al., 2010).

### Ectonucleotidase Inhibitors

Nucleotide signaling through P2X and P2Y receptors is predominantly modulated through the hydrolysis of the extracellular nucleotides by plasma membrane bound ecto-nucleotidases that have an extracellular active site. The ecto-nucleotidases are divided in four classes. The first family include the ectonucleoside triphosphate diphosphohydrolases (E-NTPDases). The E-NTPDases are nucleotide-specific and hydrolyze nucleoside triphosphates and diphosphates yielding the corresponding nucleoside monophosphates. Eight isoenzymes (NTPDase1-8) have been identified in mammals and they represent the major nucleotide-hydrolyzing enzymes involved in purinergic signaling. The second group is an ecto-5′-nucleotidase, also known as CD73, which hydrolyzes AMP to adenosine. Nucleotide pyrophosphatase/phosphodiesterase (NPP) enzymes constitute the third class. The seven isozymes of this family are capable of hydrolyzing phosphate esters of different substrates ranging from nucleotides to lipids. Among these isozymes, NPP1, NPP3, NPP4, and NPP5 are the only isozymes that act on nucleotide substrates. Finally, alkaline phosphatases finally hydrolyze nucleoside tri-, di-, and monophosphates, pyrophosphate and a large variety of additional monoesters of phosphoric acid. In this review, only ecto-nucleotidases for which inhibitors based on a nucleoside phosphonate scaffold are known are discussed.

#### NTPDase Inhibitors

**Figure 12:**
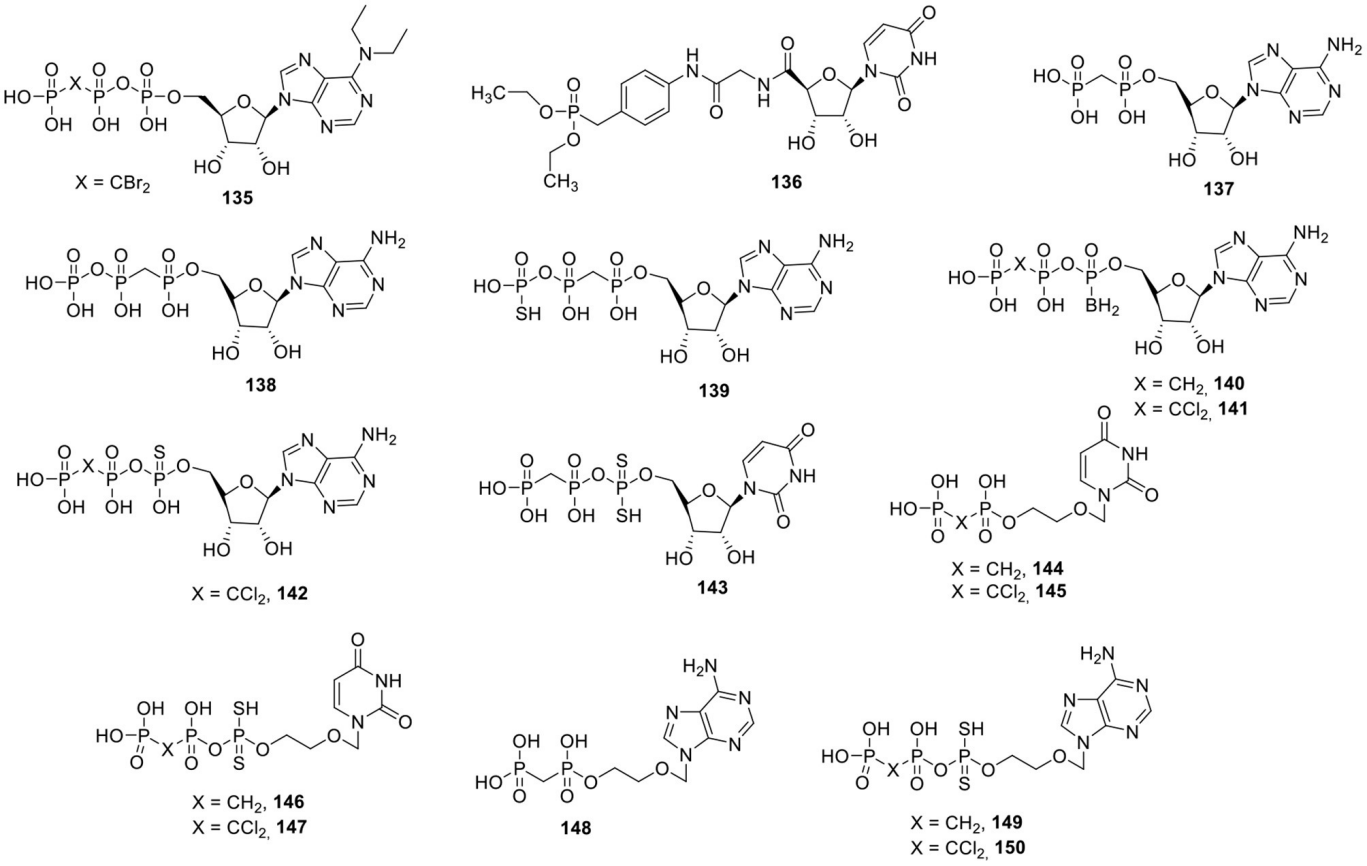
NTPDase and NPP1 inhibitors.

6-*N,N*-diethyl-β,γ-dibromomethylene adenosine triphosphate (also known as ARL 67156, compound **135**) is a weak inhibitor of NTPDase1 (CD39) and NTPDase3, with Ki values of 11 and 18 μM, respectively (Figure 12) (Levesque et al., 2007). In an effort to obtain novel, drug-like NTPDase 2 inhibitors, the triphosphate group of the natural nucleoside triphosphates was replaced by a phosphonic acid diester moiety linked via an amide bond and a linker to the nucleoside core. The most potent compounds that emerged from this series (compound **136**) was endowed with a Ki value of 8.2 μM for NTPDase2, and lacked activity against NTPDase1 and 3.

#### NPP1 Inhibitors

Although several non-nucleotide NPP inhibitors are known in literature, the best studied inhibitors of NPP1 are substrate analogs (Figure 12) (Lee and Muller, 2017). Adenosine 5′-(α,β-methylene)diphosphate (compound **137**) and adenosine 5′-(α,β-methylene)triphosphate (compound **138**) are both weak inhibitors of NPP1 (Ki values of 16.5 and 3.32 μM, respectively, when evaluated against ATP as the natural substrate) (Lee et al., 2017). Because of the presence of zinc ions in the active site of NPP1, a number of thiophosphate was prepared as sulfur is known to have a strong affinity for this metal. The introduction of a sulfur atom instead of an oxygen at the terminal phosphate moiety afforded compound **139**, which showed potent inhibition of the human membrane-bound NPP1 with a Ki value in the low nanomolar range (Ki = 0.02 μM), using *p*-nitrophenyl-5′-thymidine monophosphate (p-Nph-5′-TMP) as artificial substrate (Nadel et al., 2014). In addition to the insertion of metabolically stable methylene moieties, a borano group was introduced into the α-phosphate, yielding compound **140**, which, was found to inhibit human membrane-associated NPP1 with a Ki of 0.5 μM, when tested vs. p-Nph-5′-TMP as a substrate (Lecka et al., 2013). In addition, this compound has a promising selectivity profile as it lacked activity against various other ectonucleotidases (NPP3, NTPDase1-3). The corresponding dichloro-β,γ-methylene derivative **141** showed a reduced inhibitory potency (Ki = 18 μM). The insertion of a dichloromethylene linker between the β- and γ-phosphate moiety combined with a thiophosphate group yielded compound **142**, displaying a Ki value of 0.68 μM (Nadel et al., 2014).

Besides its inhibition toward NTPDase 1 and 3, 6-*N,N*-diethyl-D-β,γ-dibromomethylene ATP (ARL 67156, compound **135**) is also a weak inhibitor of human NPP1 (Ki = 12 μM against the artificial substrate p-NO_2_-Ph-TMP) (Levesque et al., 2007).

In order to study the SAR of UTP analogs as NPP-1 inhibitors, both Pα non-bridging oxygen atoms of UTP were replaced by sulfur and a methylene linker was inserted yielded compound **143** that was endowed with a NPP1 Ki value of 1.11 μM (Figure 12) (Zelikman et al., 2018). In addition, a series of acyclic phosphonates, derived from UTP, was prepared (Nassir et al., 2019b). The acyclic UDP analogs with an α,β-bridging methylene or dichloromethylene group (compounds **144** and **145**) displayed only moderate inhibitory effect on human NPP1 (54 and 51% inhibition at 100 μM, respectively). Dithio substitution on the Pα-phosphate turned these analogs into more potent NPP1 inhibitors with Ki values of 0.94 and 0.73 μM, for the methylene (compound **146**) and dichloromethylene (compound **147**) derivative, respectively.

Similarly, a series of acyclic nucleoside phosphonates carrying adenine as nucleobase was synthesized (Figure 12). Adenine-N9-(methoxy)-ethyl-β-bisphosphonate **148** was a dual NPP1/CD73 inhibitor (Ki values of 9.6 and 12.6 μM, respectively, when tested vs. the natural substrate ATP) and lacked activity against NPP3, NTPDase1, and tissue non-specific alkaline phosphatase (TNAP) (Nassir et al., 2019a). The analogs in which the α-phosphate groups was replaced by a dithiophosphate group only showed modest NPP1 inhibition, with IC_50_ values of 111 and 25.9 μM, for the methylene (compound **149**) and dichloromethylene (compound **150**) derivative, respectively, when tested vs. natural ATP as the substrate.

#### CD73 Inhibitors

**Figure 13:**
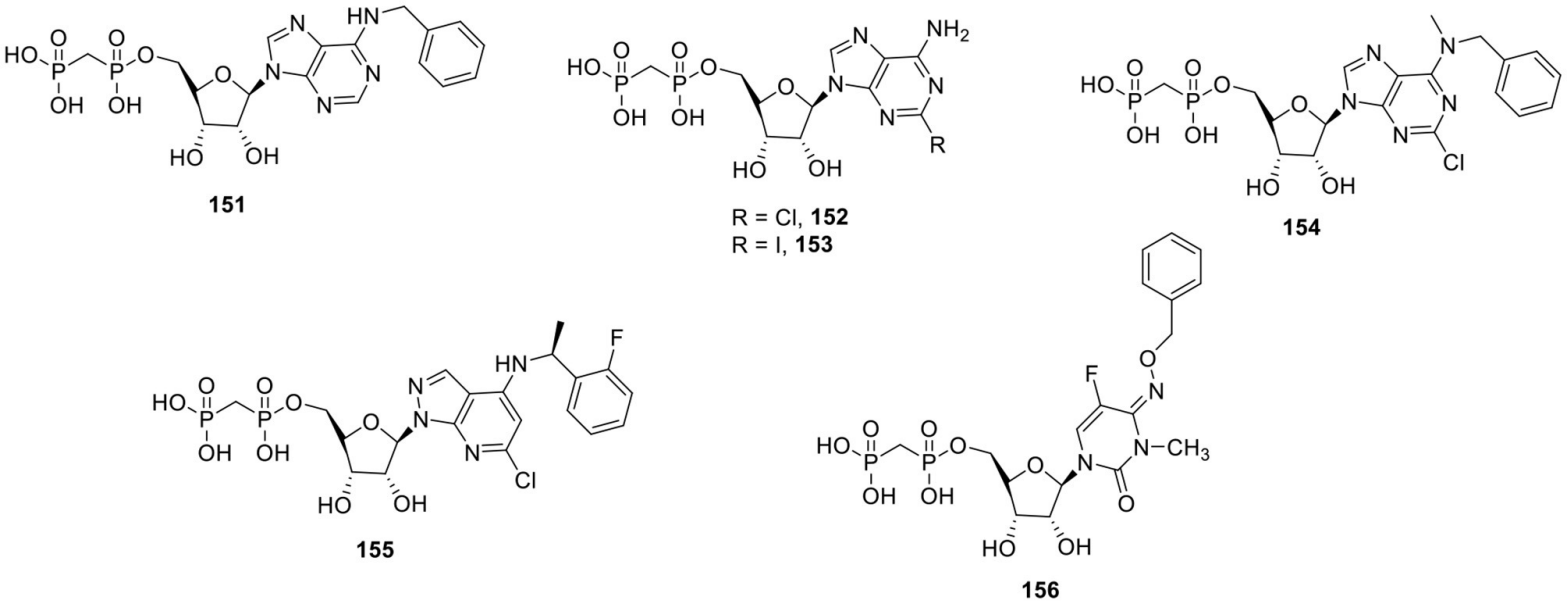
CD73 inhibitors.

Ecto-5′-nucleotidase, also known as CD73, is an ecto-5′-nucleotidase which hydrolyzes AMP to adenosine and is considered as a promising drug target for immune-oncology. The most potent CD73 inhibitors are all nucleoside-5′-diphosphate analogs (Figure 13). The ADP analog **137** is endowed with a Ki value of 88 nM for human CD73. Structural modification of the nucleobase allowed to further increase the potency. A benzyladenosine analog (compound **151**) showed a Ki value of 2.21 nM and has a promising selectivity profile as it had no activity on any other ectonucleotidases (Bhattarai et al., 2015). Structural modification at position 2 of the nucleobase was tolerated as the 2-iodo and 2-chloro-adenosine analogs (compounds **152** and **153**) were potent CD73 inhibitors, displaying Ki values of 3 and 5 nM, respectively. In addition, it was demonstrated that the 2-iodo congener was metabolically stable in the presence of human plasma and rat liver microsomes (Bhattarai et al., 2020). Combination of a 2-chloro substituent with a *N*-Me-benzyl group furnished compound **154**, a very potent CD73 inhibitor (Ki = 0.318 nM) (Bhattarai et al., 2019). Nucleotide analogs with a nucleobase that deviate more from the natural adenine base are also known. An example includes compound **155**, that had a pyrazolo[3,4-*b*]pyridine nucleobase. The presence of a 2-chloro and a 6-benzylamino substituent made this compound a very potent CD73 inhibitor, displaying a Ki value of 0.005 nM (Bowman et al., 2019). Besides purine nucleoside phosphonates, pyrimidine nucleoside phosphonates, are also known to act as potent CD73 inhibitors. The synthesis of a series of N4-substituted-cytosine derivatives afforded potent CD73 inhibitors. Especially analogs carrying a bulky benzyloxy substituent (as exemplified by compound **156**) were potent and selective CD73 inhibitors (Ki = 7.96 nM).

## Conclusions and Perspectives

The heterogeneous key roles played by naturally occurring nucleoside phosphates in numerous biochemical processes has stimulated medicinal chemists to design nucleoside phosphonates as their isosteric and isopolar counterparts with increased resistance toward *in vivo* enzymatic degradation. Over the past 40 years, this concept has been the subject of extensive synthetic efforts that resulted in the marketing of a limited number of nucleoside phosphonate drugs. The most noteworthy examples are cidofovir, adefovir, and tenofovir that received approval for the clinical treatment of different viral infections. In addition, a variety of other nucleoside phosphonate analogs, both cyclic as well as acyclic, with excellent activity against retroviruses and/or DNA viruses have been described in the literature. However, an in-depth, side-by-side comparison with the existing agents is required to identify competitive advantages in terms of efficacy, toxicity, and/or resistance profile in order to justify their further development as antiviral drugs. Nucleoside phosphonate analogs with potent activity against RNA viruses remain elusive up to now. As current outbreaks of infectious diseases are mainly caused by RNA viruses, such as the Ebola virus (EBOV) and severe acute respiratory syndrome coronavirus 2 (SARS-CoV2), there is a huge interest in the discovery of broad-spectrum antiviral agents based on a nucleoside phosphonate scaffold that may be active against various RNA viruses.

The application of nucleoside phosphonate chemistry in other therapeutic areas has been lagging behind in comparison to the antiviral scene. Outside the antiviral area, only two nucleoside phosphonates were licensed for medical use. Specifically, Rabacfosadine received marketing approval by the FDA for the treatment of lymphoma in dogs, whereas Cangrelor was licensed for clinical use as an antiplatelet drug.

The main drawbacks associated with nucleoside phosphonates are their high polarity and consequentially limited cellular permeability as well as poor oral bioavailability. In the antiviral/antitumoral area, these hurdles were overcome with the synthesis of a variety of prodrugs. Similar promoieties were used to derivatize nucleoside phosphonate analogs that showed promising biochemical potency against isolated bacterial or parasitic targets. Penetration of small molecules through the bacterial membrane (especially for Gram-negative pathogens) remains in fact a major challenge in antibacterial drug discovery. However, it is likely that prodrugs that are optimized for antiviral and antitumoral drug discovery are not optimal for antibiotics, therefore highlighting the need for the development of new prodrug strategies specifically designed for bacterial drug delivery.

Several nucleoside phosphonates acting as agonists/antagonists of various purinergic receptors or ecto-nucleotidase inhibitors are known; however, only a few showed potent activity. In addition, selectivity between the different receptors and/or enzymes is still an issue for several of these compounds. Therefore, an expanded chemical space needs to be explored to gain access to analogs with potency in the nanomolar range and an acceptable selectivity window before these compounds can be used as pharmacological tools. Moreover, prior to use these compounds in preclinical animal models, they will have to be converted to a suitable prodrug.

Nonetheless, the continuously increasing awareness of the most influential factors that needs to be taken into consideration in this research field can be reasonably expected to sustain the future translation of fundamental research into new nucleoside phosphonate drugs.

## Author Contributions

All authors listed have made a substantial, direct and intellectual contribution to the work, and approved it for publication.

## Conflict of Interest

The authors declare that the research was conducted in the absence of any commercial or financial relationships that could be construed as a potential conflict of interest.
